# Responsiveness of Early Response to Dehydration Six-Like Transporter Genes to Water Deficit in *Arabidopsis thaliana* Leaves

**DOI:** 10.3389/fpls.2021.708876

**Published:** 2021-08-16

**Authors:** Lucie Slawinski, Abir Israel, Caroline Artault, Florence Thibault, Rossitza Atanassova, Maryse Laloi, Fabienne Dédaldéchamp

**Affiliations:** Laboratoire Ecologie et Biologie des Interactions, UMR Centre National de la Recherche Scientifique 7267, Université de Poitiers, Poitiers, France

**Keywords:** sugar transporters, monosaccharides carriers, ERD6-like, ESL, water deficit, arabidopsis leaves, *AtESL* mutants

## Abstract

Drought is one of the main abiotic stresses, which affects plant growth, development, and crop yield. Plant response to drought implies carbon allocation to sink organs and sugar partitioning between different cell compartments, and thereby requires the involvement of sugar transporters (SUTs). Among them, the early response to dehydration six-like (ESL), with 19 members in *Arabidopsis thaliana*, form the largest subfamily of monosaccharide transporters (MSTs) still poorly characterized. A common feature of these genes is their involvement in plant response to abiotic stresses, including water deficit. In this context, we carried out morphological and physiological phenotyping of *A. thaliana* plants grown under well-watered (WW) and water-deprived (WD) conditions, together with the expression profiling of 17 *AtESL* genes in rosette leaves. The drought responsiveness of 12 *ESL* genes, 4 upregulated and 8 downregulated, was correlated to different water statuses of rosette leaves. The differential expression of each of the tandem duplicated *AtESL* genes in response to water stress is in favor of their plausible functional diversity. Furthermore, transfer DNA (T-DNA) insertional mutants for each of the four upregulated *ESLs* in response to water deprivation were identified and characterized under WW and WD conditions. To gain insights into global sugar exchanges between vacuole and cytosol under water deficit, the gene expression of other vacuolar SUTs and invertases (*AtTMT, AtSUC, AtSWEET*, and *At*β*FRUCT*) was analyzed and discussed.

## Introduction

The increase of crop productivity face to climate change is a major societal challenge. Yield losses due to abiotic constraints, in an example of water deficit, range from 50 to 80% and even exceeded those caused by biotic stresses. In terms of fundamental research, drought responses of model plants and crops have long been studied for the purpose of improvement and/or survival under the conditions of severe water deficit (SD). However, this tolerance/survival predicts neither better growth capacity nor the enhancement of productivity. In the last decade, the global vision of research focused on genes, which results in the elevation of biomass and the yield of seeds under the conditions of moderate water stress (Skirycz et al., 2011; Raza et al., 2019; Zhao et al., 2020). To cope up with water deficit, plants have developed different strategies such as escape, avoidance, and osmotic adjustment strategies (Arve et al., 2011; Fang and Xiong, 2015; Ilyas et al., 2020). The escape strategy deals with the completion of the plant life cycle at the onset of drought, which reflects a high degree of developmental plasticity (Chaves et al., 2003; Lawlor, 2013; Marín-de la Rosa et al., 2019). The avoidance strategy deals with the ability of plants to grow during periods of drought and to maintain their water status by: (1) the limitation of water loss by reducing leaf surface area and stomatal conductance (SC), and, by consequence, transpiration; (2) the optimization of water supply with deeper roots, which requires the reallocation of photoassimilates in favor of root growth, thereby increasing their water absorption capacity; and (3) the storage of water in specialized organs (Griffiths and Males, 2017). The osmotic adjustment strategy is used by plants to cope up with water deficit by accumulating compatible osmolytes (proline, sucrose, polyols, etc.) to maintain the water potential of the cells and also the turgor pressure to support their growth (Hare and Cress, 1997; Hare et al., 1998; Xiong and Zhu, 2002; Moore et al., 2008; Lawlor, 2013; Ilyas et al., 2020). In this context, the accumulation of soluble sugars has been observed in the leaves of *Arabidopsis thaliana* during water deficit (Taji et al., 2002; Hummel et al., 2010; Mewis et al., 2012; Sperdouli and Moustakas, 2012). Under mild and moderate water deficit (MD), SC decreases, but a partial closure of stomata does not prevent photosynthesis activity. However, the efficiency of photosynthesis is reduced under more severe water stress (Lawlor, 2002; Lawlor and Cornic, 2002; Muller et al., 2011; Zhao et al., 2020).

In plants, subcellular sugar partitioning and storage require the activity of sugar transporters (SUTs) located on diverse membranes especially vacuoles (Hedrich et al., 2015). SUTs are classified into three main families: sucrose transporters/SUTs (SUCs/SUTs), sugar will eventually be exported transporters (SWEET), and monosaccharide transporters (MSTs). SUC/SUT carriers are mainly H^+^/sucrose symporters localized on the plasma membrane (Meyer et al., 2004; Sauer, 2007; Sivitz et al., 2007, 2008; Reinders et al., 2012), except SUT4 carriers (Type III SUTs), which are targeted to the tonoplast (Endler et al., 2006; Reinders et al., 2008, 2012; Eom et al., 2011). The SWEET family has been more recently identified. The family members characterized as uniporters are involved in the flux of sucrose, glucose, and/or fructose (Chen et al., 2010; Eom et al., 2015; Jeena et al., 2019). AtSWEET2, AtSWEET16, and AtSWEET17 have been described as vacuolar facilitators with a predominant expression in root vacuoles (Guo et al., 2014). AtSWEET2 has been described to transport the glucose analog 2-deoxyglucose. It is expressed in the cortex and epidermis and is probably involved in the sequestration of glucose in root vacuoles, thereby preventing sugar secretion into the rhizosphere (Chen et al., 2015). AtSWEET16 transports glucose, fructose, and sucrose; it is expressed in vascular parenchyma cells and is downregulated by sugars, cold, osmotic stress, and low nitrogen. AtSWEET16 overexpression improves germination, growth efficiency, and tolerance against freezing temperatures (Klemens et al., 2013). *AtSWEET17* has first been described as a leaf vacuolar exporter specific for fructose (Chardon et al., 2013). It is also strongly expressed in root cortex cells, by acting as a bidirectional fructose facilitator to mediate fructose uptake into the vacuole for the storage or export of fructose to the cytosol to maintain cell metabolism (Guo et al., 2014). The family of MSTs is divided into seven subfamilies. Three of them have the members that are located on the tonoplast: tonoplastic monosaccharide transporters/tonoplast sugar transporters (TMTs/TSTs), vacuolar glucose transporters (VGTs), and early response to dehydration six-like (ESL) (Büttner, 2007; Slewinski, 2011). AtTMT1 and AtTMT2 have been suggested to function as H^+^/glucose antiporters (Wormit et al., 2006) and later on have also been characterized as H^+^/sucrose antiporters (Schulz et al., 2011). Expressed in most plant tissues, they are regulated by sugars and abiotic stresses such as cold, salt, and drought. The latter is corroborated in TMT1/TMT2 knockout lines, which have shown reduced capacity to accumulate glucose and fructose under cold stress (Wormit et al., 2006). The overexpression of TMT1 in *A. thaliana* modifies the subcellular sugar compartmentation and increases sugar export capacity from the source to sink tissue leading to improved seed production (Wingenter et al., 2010). AtVGT1, the only characterized member of this subfamily, is expressed at low levels in green tissues and strongly in pollen. It has been localized on the tonoplast and described to be involved in an energy-dependent transport of glucose and fructose. *atvgt1* knockout mutants show a decrease in seed germination and a delay in flowering, which imply the involvement of this transporter in developmental processes (Aluri and Büttner, 2007). In *A. thaliana*, 19 ESL transporters have been identified. Although they constitute the largest MST subfamily in *Arabidopsis*, their role is poorly understood. To get insights into this issue, Slawinski et al. (2021) applied an evolutionary genomics approach to study ESL transporters in the genome of 63 species representative of the plant kingdom, from algae to angiosperms, and suggested that these transporters might have emerged from a common streptophyte ancestor, and have evolved through diversification events and tandem duplications. Phylogenetic analysis of the 519 identified protein sequences showed that these ESL transporters belong to three subgroups, ESL1, ESL2, and ESL3, and based on their phylogenetic position, a new nomenclature has been suggested to reflect their evolution. The expression profiling of *Arabidopsis ESL* genes in different organs (leaves, roots, flower buds, flowers, and siliques) has shown that *ESL1* and *ESL2* genes are, respectively, moderately and weakly expressed, whereas *ESL3* display a high expression diversity. This study has demonstrated differential expression patterns between the copies of the *ESL* tandem pairs, suggesting a functional diversification of these transporters (Slawinski et al., 2021).

Because the identification of the first *ESL* gene (*ESL3.08/ERD6 – At1g08930*) *via* differential screening of a complementary DNA (cDNA) library prepared with *A. thaliana* plants exposed to dehydration (Kiyosue et al., 1994), only few ESL genes have been characterized. These transporters are involved in plant development and responses to various abiotic stresses. In 4-week-old *A. thaliana* plants, the level of *AtESL3.08*/*ERD6* messenger RNA (mRNA) seems very low but is transiently increased in response to dehydration and cold (4°C) (Kiyosue et al., 1998). *AtESL3.08*/*ERD6* expression in leaves and roots is similar under normal growth conditions, but decreases in leaves under high salinity (250 mM NaCl) conditions and after 100 μM abscisic acid (ABA) treatment (Yamada et al., 2010). At present, *AtESL3.08/ERD6* is considered to take part in the redistribution of sugars, thereby protecting plant cells from the detrimental effects of dehydration and cold stresses (Kiyosue et al., 1998).

*AtESL3.07*/*ESL1 (ERD six-like-1—At1g08920)* is a tandem duplicated gene of *AtESL3.08*/*ERD6* and are located on chromosome 1. In 3-week-old *A. thaliana* plants, *AtESL3.07*/*ESL1* expression is induced after 1-h treatment by dehydration, high salinity (250 mM NaCl), and ABA (100 μM). Without stress, this expression is higher in roots than in leaves and it is highly induced in roots under high salinity and ABA supplementation. At*ESL3.07/ESL1* protein has been described as a low-affinity facilitator capable of transporting different hexoses such as glucose, fructose, galactose, mannose, and xylose (Yamada et al., 2010).

*AtESL3.13/AtSFP1* and *AtESL3.14/AtSFP2* (sugar-porter family protein 1 and 2—*At5g27350* and *At5g27360*) are the two tandem duplicated genes located on chromosome 5. These two genes display different expression patterns during leaf development. *AtESL3.13/AtSFP1* is detected in seedlings 9 days after germination but not in mature plant organs (leaves, flowers, flower buds, stem, and roots), while *AtESL3.14/AtSFP2* is expressed in all tested organs and in seedlings. Further analysis shows that only *AtESL3.13/AtSFP1* is induced during leaf senescence while the expression of *AtESL3.14/AtSFP2* remains stable during this process (Quirino et al., 2001).

*AtESL1.02/ERDL6* (early responsive to dehydration-like six—*At1g75220*) is a vacuolar H^+^/glucose symporter involved in the export of glucose from the vacuole to cytosol (Klemens et al., 2014). This expression is regulated by the mobilization of vacuolar carbohydrate reserves: an upregulation by darkness, heat stress, and wounding and a downregulation under cold stress and glucose supply. *atesl1.02* mutant presents a higher vacuolar glucose content (>90%) than the wild-type mutant (86%), and cell lines overexpressing *ESL1.02*/*ERDL6* have glucose content lower than that of wild plants. These data indicate that ESL1.02/ERDL6 would act as an exporter of vacuolar glucose during developmental phases with a high metabolic turnover, such as seed germination, abscission zones, and wound responses (Poschet et al., 2011). In apple, MdERDL6-1, a homolog to ESL1.02/ERDL6 in *Arabidopsis*, has recently been identified. It is highly expressed in fruits, and its encoded protein seems to be localized on the tonoplast, where it acts as a low-affinity H^+^/glucose symporter. In an inverse relation to AtERDL6, the overexpression of MdERDL6-1 in apple and tomato leaves and fruits leads to an increase rather than a decrease in sugar levels (Zhu et al., 2021).

*AtESL2.01/AtZIF2* (zinc-induced facilitator—At2g48020) is a tonoplastic transporter involved in the vacuolar sequestration of zinc at the root level. Transporter function loss causes hypersensitivity to Zn, whereas its overexpression increases tolerance (Remy et al., 2014). Until now, *AtESL2.01/AtZIF2* capacity of transporter to transport sugars is not demonstrated.

More recently, Desrut et al. (2020) have shown that six *AtESL* genes are differentially regulated in the presence of plant growth-promoting rhizobacteria (PGPR): *ERD6-like7, ERD6-like12*, and *ERD6-like16*, as well as *ERD6-like18*, are downregulated in shoots and roots, respectively, whereas *ERD6-like13* and *ERD6-like15* are upregulated in roots in the response of PGPR.

Furthermore, Breia et al. (2020) have shown that the grapevine *VvERD6l13* is induced in the grape berries upon infection by necrotrophic or biotrophic pathogens such as *Botrytis cinerea* and *Erysiphe necator*. VvERD6l13 is the first ESL described to be localized in the plasma membrane as demonstrated in tobacco epidermal cells. More surprisingly, using yeast as a heterologous system, VvERD6l13 has been characterized as a low-affinity H^+^/sucrose symporter.

In this study, we characterized the integrative physiological response of *A. thaliana* Col-0 plants to gradual water deprivation and established the following phases of the water status of rosette leaves, i.e., “early mild water deficit” (EM), “MD,” “SD,” and “wilting” (Wi). To explore the involvement of *AtESL* genes in plant response to water deficit, we evaluated growth, physiological, and biochemical parameters in parallel with the levels of *AtESL* expression. Furthermore, transfer DNA (T-DNA) insertional mutants for the four *ESL* genes upregulated in response to water deficit were identified, and characterized under well-watering, water deprivation, and re-watering. To get global insights into sugar exchanges during water deficit between two subcellular compartments, vacuole, and cytosol, the expression profiles of known SUTs located on the tonoplast (*AtTMT, AtVGT, AtSUC*, and *AtSWEET*) in leaf cells were established, as well as those of two vacuolar invertases (*At*β*FRUCT*).

## Materials and Methods

### Plant Materials

*Arabidopsis thaliana*, wild-type Col-0 and the following T-DNA insertion lines: SALK_106049 (*at1g75220-esl1.02/erdl6*), SALK_132009 (*at4g04760-esl3.03*), SALK_047351 (*at1g08890-esl3.05/esl3*), and SALK_025646 (*at1g08920*-*es3.07/esl1*) were used in this study (Supplementary Table 1). T-DNA single-insertion mutant homozygote lines were identified *via* a SIGnAL site (SALK Institute Genomic Analysis Laboratory, La Jolla, CA, USA) and provided by NASC (the European Arabidopsis Stock Center).

### Growth Conditions

The seeds of Col-0 and SALK-mutants were sown in pots containing a sterile mixture of soil:vermiculite (3:1, w/w) and placed in darkness for 2 days at 4°C. Such a stratification reduces residual seed dormancy and improves seed germination. Afterwards, pots were placed in a growth-controlled chamber with the following parameters: 10-h day at 100 μmol.m^−2^.S^−1^ of light intensity/14-h night, 22/18°C (day/night), and 50/90% (day/night) relative humidity. After 13 days, the seedlings were individually transplanted into Arasystem^TM^ (BETATECH bvba, Gent, Belgium) completed with 50 ± 2 g of soil: vermiculite mixture (3:1). Until the 35th day of growth, the plants were watered once per week and supplemented with a fertilizer (PETERS: 20/20/20; N/P/K).

**Figure 1:**
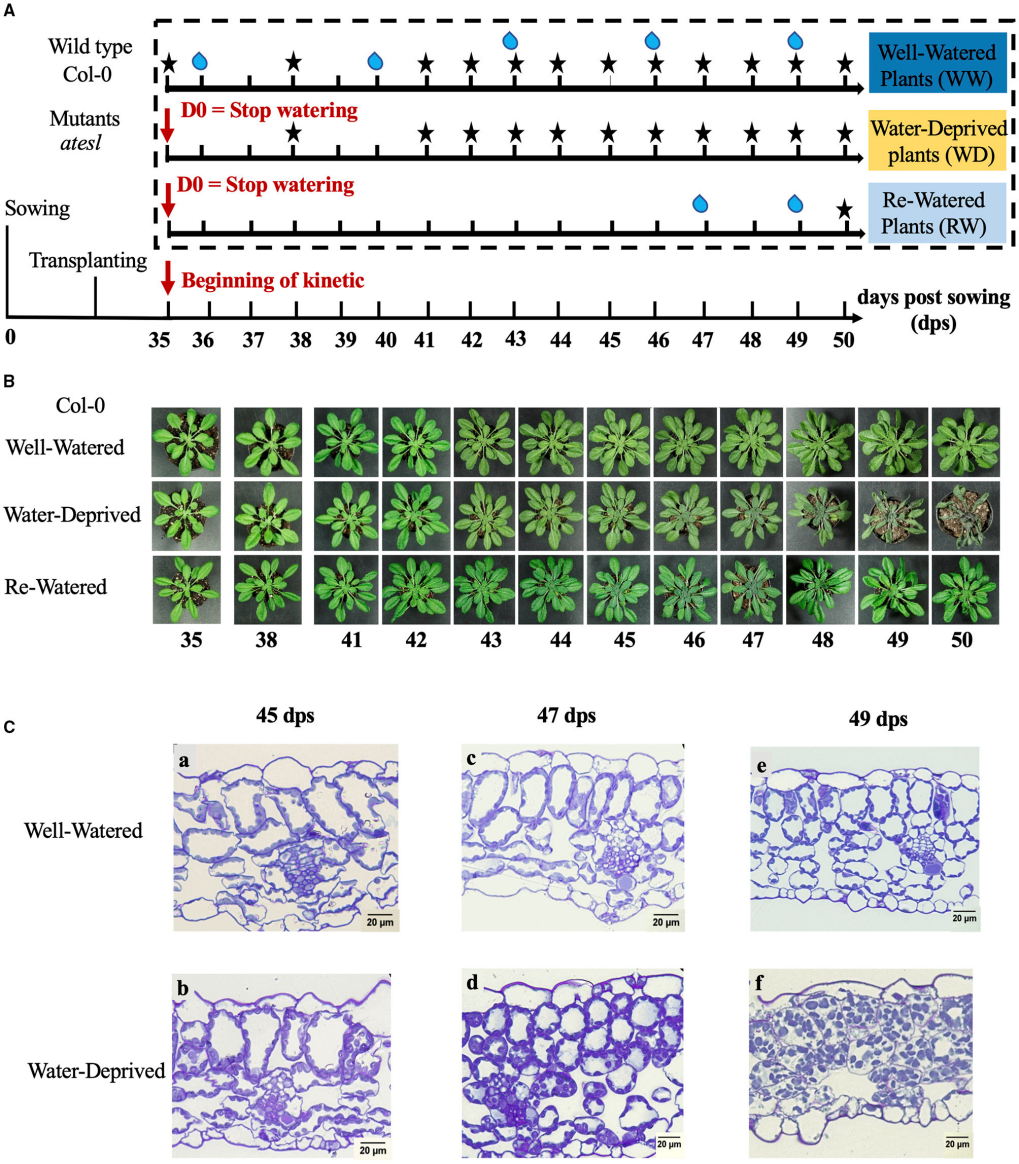
Growth conditions, rosette phenotype, and leaves under microscopy observations of *Arabidopsis thaliana*. **(A)** Schematic representation of the different culture conditions of Col-0 and *atesl* mutants. WW, WD, and RW stand for well-watered, water-deprived, and re-watered, respectively. The days of watering are indicated by bleu drops and of harvest by black asterisks. **(B)** Development of Col-0 rosettes in WW, WD, and RW conditions during the 15 days of growth kinetics. **(C)** Light microscopy of Col-0 leaves cross-sections at the 45th (a,b), 47th (c,d), and 49th day post-sowing (dps) (e,f). The study was carried out with 10 plants per condition, and 3 independent biological repeats were performed (±SD).

To characterize the effect of water deficit on Col-0 and *atesl1.02/erdl6, atesl3.03, atesl3.05/esl3*, and *atesl3.07/esl1* mutants, the seeds were cultivated for 35 days as described above. On the 35th day of post-sowing (dps), one batch of plants were watered every 3 days [well-watered (WW) plants], a second batch of plants were not watered for 15 days [water-deprived (WD) plants], a third batch of plants were not watered until the 12th day (47th dps) and later on re-watered (RW) (Figure 1A). The experiment was repeated independently six times, with 12 sampling time points (35, 38, and 41 dps and then each day until 50 dps). Each time point corresponded to the average of five plants. For each sampling, one half of a rosette was used to measure physiological parameters, and another half was frozen in liquid nitrogen for further RNA and sugars extractions.

For ABA treatment, the rosettes of 43-day-old Col-0 plants were sprayed with ABA solution (750 μM ABA in 0.75% DMSO and 0.02% Triton X-100). Leaves were collected immediately (T0) and every 2 h during 8 h and frozen in liquid nitrogen for further RNA purification. The leaves of rosettes were sprayed with a DMSO solution (0.75% DMSO and 0.02% Triton X-100) and the untreated leaves were used as control.

### Growth and Physiological Parameters

#### Leaf Water Status

Each rosette was divided diametrically into two equivalent parts, which therefore contain the same proportion of leaves at different development stages. Each part was weighed immediately after harvest. The total fresh weight was calculated by the addition of the two weights. One half of a rosette was placed in distilled water for 24 h, at 4°C, under darkness, then a turgid weight (TW) was measured and a rosette material was dried for additional 24 h at 80°C to determine dry weight (DW). Based on these measurements, the total TW and DW were calculated by relating it to the total mass. The relative water content (RWC) and water content (WC) were calculated as RWC = (FW–DW)/(TW–DW) and WC = (FW–DW)/FW. The second half of a rosette after weighing was immediately frozen in liquid nitrogen until further analysis of such gene expression and sugar content.

#### Projected Leaf Area and SC

The projected leaf area (PLA) of a photographed rosette was determined using the software ImageJ (Schneider et al., 2012). Five plants per genotype and treatment were followed during the 15 days of experimentation. SC (mmol/m^2^/s) was measured using a leaf porometer (Model SC-1, Decagon Devices Inc., Pullman, WA, USA). Measurements were taken daily after an illumination period of 5 h. Two measurements were taken per plant (i.e., two leaves per plant), and 10 plants per genotype and per condition were used.

#### Content of Soluble Sugars

The extraction of soluble sugars was carried out using the half of rosette previously ground in liquid nitrogen (TissueLyser II, Qiagen, Hilden, Germany) and lyophilized. Approximately 10 mg of powder was extracted by three washings (one time with 1.5 ml and two times with 0.5 ml) in methanol:chloroform:water (12:5:3, v/v/v), then the supernatants containing soluble sugars were pooled and mixed with distilled water at a final volume ratio of 0.6. After the centrifugation at 1,200 g for 15 min, the upper phase was collected and vacuum dried at 50°C for 3 h in MiVac Quattro (Genevac, Ipswich, UK). The soluble sugars were quantified by using the Suc/Fru/D-Glc Assay Kit (Megazyme, County Wicklow, Ireland) according to the instructions of the manufacturer.

#### Osmotic Pressure

To determine the osmotic pressure of the cells, the leaf fragments without the main vein were weighed immediately after harvest. After an incubation for 24 h, at 4°C in sucrose solutions with molarity ranging from 0.01 to 1 M, leaf fragments were weighted. Three plants by genotype and by growth condition were used at 45, 47, and 49 dps.

#### Microscopy Analysis

Leaf samples were immediately fixed for 1 h, at 4°C in a solution mixture of 2% paraformaldehyde, 0.5% glutaraldehyde, and 0.05 M phosphate buffer, pH 7.2 (v/v/v). After three washes for 20 min each in 0.2 M phosphate buffer and pH 7.2 supplemented with 7.5% sucrose, a post-fixation step in 1% osmium tetroxide for 5 min followed. The dehydrated samples were embedded in LR White resin according to Fleurat-Lessard et al. (2016). Sections were made using a microtome (EMUC6, Leica, Wetzlar, Germany), stained with toluidine blue and observed through light microscopy (Zeiss Axioplan, Berlin, Germany). Three plants by genotype and by growth conditions were used at 45, 47, and 49 dps.

### Molecular Biology

#### DNA Extraction and ESL Mutant Lines Screening

Genomic DNA was extracted using the NucleoSpin® Plant II Kit (Macherey-Nagel, Düren, Germany) following the instructions of the manufacturers. The presence of T-DNA inserts and the homozygosity of mutant lines were confirmed by PCR analysis using two gene-specific primer (LP and RP) to amplify the wild-type allele and LBb1.3 (T-DNA primer) with RP or LP (T-DNA primer) to amplify a mutant allele. All primer sequences are shown in Supplementary Table 1.

#### RNA Extraction and cDNA Synthesis

The frozen leaf samples were ground using TissueLyser II QIAGEN (Hilden, Germany) and subsequently, the total RNA was extracted as described by Kay et al. (1987). RNA was quantified in a Nanodrop 1000 spectrophotometer (Thermo Scientific, Waltham, MA, USA), and RNA integrity was checked by 1.5% (w/v) agarose gel electrophoresis. cDNA was synthetized from 1 μg of total RNA after DNAse treatment (Sigma-Aldrich, St. Louis, MO, USA) using MML-V reverse transcriptase (Promega, Madison, WI, USA).

#### Quantitative PCR Analysis

The expression analysis of the 17 *AtESL*: *AtESL1.01* (At1g19450), *AtESL1.02/ERDL6* (At1g75220), *AtESL2.01/ZIF2* (At2g48020), *AtESL2.02* (At3g5150), and *AtESL2.03* (At5g18840), *AtESL3.01* (At1g54730), *AtESL3.02* (At4g4750), *AtESL3.03* (At4g04760), *AtESL3.04* (At3g20460), *AtESL3.05/ESL3* (At1g08890), *AtESL3.06/ESL2* (At1g08900), *AtESL3.07/ESL1* (At1g08920)*, AtESL3.08/ERD6* (At1g08930)*, AtESL3.10* (At3g05400), *AtESL3.11* (At3g05160), *AtESL3.13/SFP1* (At5g27350), and *AtESL3.14/SFP2* (At5g27360) of other vacuolar SUTs: *AtSWEET16* (At3g16690), *AtSWEET17* (At4g15920), *AtTMT1 (At1g20840), AtTMT2 (At4g35300), AtSUC4* (At1g09960), and *AtVGT2* (At5g17010), and of vacuolar invertases *At*β*Fruct3* (At1g62660) and *Atfruct4* (At1g12240) were performed using the rosette leaves of Col-0 and mutant plants grown in WW, WD, and RW conditions. Quantitative PCR (qPCR) assays were performed using the Master Cycler Realplex2 (Eppendorf, Hamburg, Germany), in a reaction of 15 μl: 5 μl of diluted cDNA as a template, 7.5 μl of SYBRGREEN® GoTaq qPCR Master Mix (Promega, Madison, WI, USA), 0.5 μl of each gene-specific primer (10 mM), and 1.5 μl of water. The qPCR program was set at 95°C for 2 min, then 40 cycles of 15 s at 95°C, 1 min at 60°C. At the end of amplification, a melting curve was generated to check the specificity of the primers (95°C, 15 s; 60–95°C, 20 min; 95°C 15 s). *AtPP2a* (At1g13320) (Czechowski et al., 2005) was used as a reference gene to normalize gene expression. The relative expression was determined according to the 2^−ΔCt^ method. Each biological replicate was tested in three technical repetitions. The primer sequences used are specific to individual transcripts, including those for tandem duplicated genes, and are presented in Supplementary Table 1.

### Statistical Analysis

Nonparametric Mann–Whitney test was used for two groups of variables. For three or more than three groups of variables, the Kruskal–Wallis and Dunn test were applied using the XLStats 2011 software (Addinsoft, Paris, France).

## Results

### Phenotype and Physiological Parameters of *A. thaliana* Plant

Phytotron-grown 35-day-old *A. thaliana* plants were subjected either to water deficit by totally withholding the water supply during 15 days (referred to as “WD”) or to 12 days of water deprivation followed by watering on the last 3 days (referred to as “RW”). In parallel, control plants were normally watered during the same period of 15 days (referred to as “WW”) (Figure 1A). Growth and physiological parameters of plants under the abovementioned conditions were compared between 35 and 50 dps, initially every 3 days (35, 38, and 41 dps), and later on daily until 50 dps. For WD plants, Wi was observed 12 days post-watering arrest (12 dpwa: 47 dps), and re-watering at 47 dps restored the WW phenotype (Figure 1B).

The microscopy analysis of thin sections of leaves showed that in control WW plants with normal macroscopic phenotypes any histological alteration was observed during the entire experimental period from 45 to 49 dps (Figure 1C). Epidermal and mesophyll cells were turgid as demonstrated by chloroplasts positioning close to the plasma membrane and cell wall. Moreover, intercellular areas were visible and well-delimited (Figures 1Ca,c,e). The original, unaltered histology was observed for WD plants at 45 and 47 dps (Figures 1Cb,d). On the contrary, at 49 dps, chloroplasts were scattered into the cells, due to the reduced vacuolar volume, on the onset of cell plasmolysis (Figure 1Cf). The loss of turgor pressure led to the reduction of intercellular areas as well as the retraction of the plasma membrane in mesophyll cells, which reflects the advanced stage of Wi of leaves (Figure 1B).

**Figure 2:**
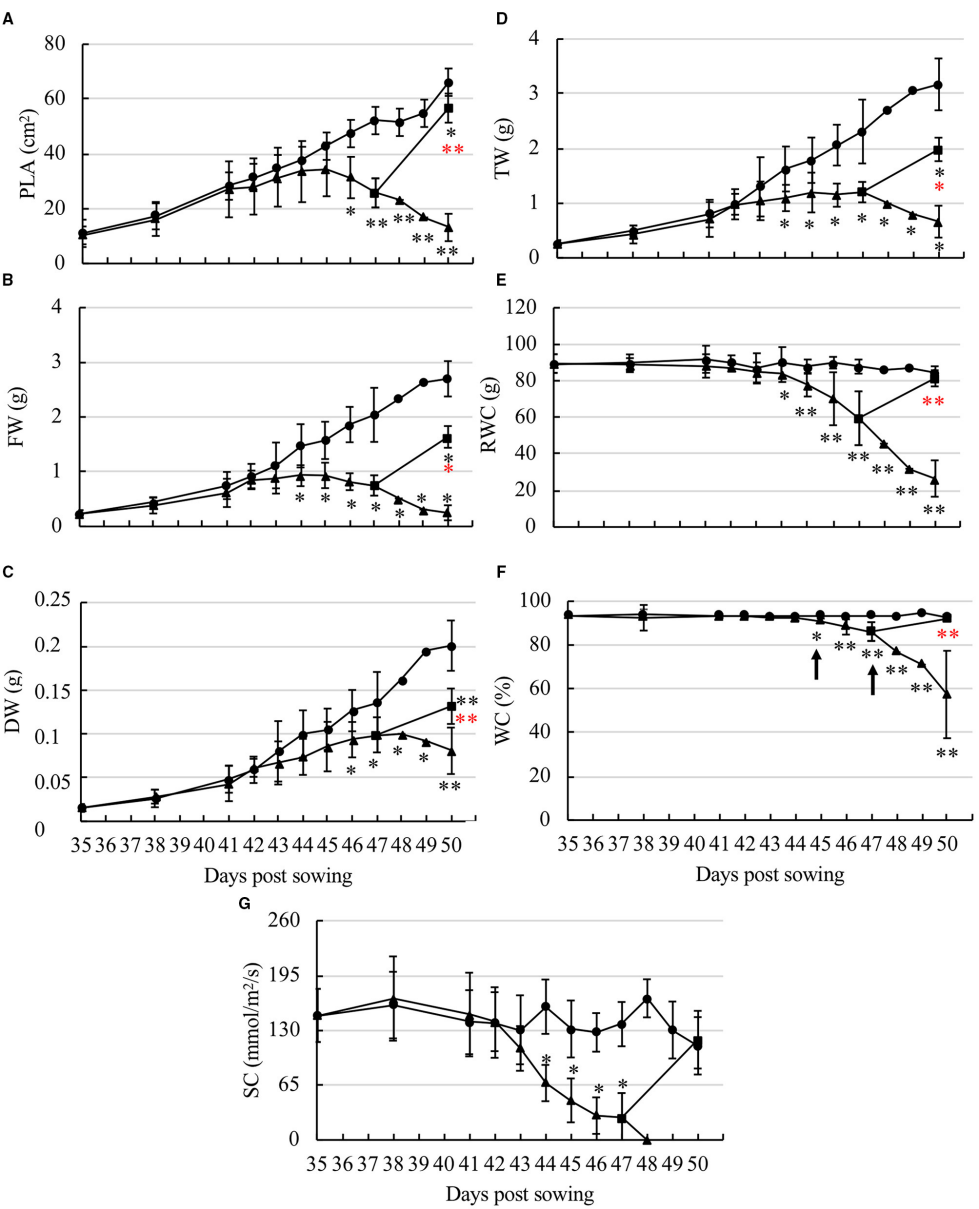
Characterization of the effect of water deficit of Col-0 *A. thaliana* leaves. **(A)** PLA, projected leaf area; **(B)** FW, fresh weight; **(C)** DW, dry weight; **(D)** TW, turgid weight; **(E)** RWC, relative water content; **(F)** WC, water content; **(G)** SC, stomatal conductance, were measured under WW (circle), WD (triangle), and RW (square). The study was carried out with five plants per condition, and three independent biological repeats were performed (±SD). Statistical analysis was performed using the Mann–Whitney pairwise comparison test (*p < 0.05, **p < 0.001). The WD values that are significantly different in comparison to WW are indicated by a black asterisk and the WD values that are significantly different in comparison to WD are indicated by a red aster.

To correlate phenotype and microscopy observations with plant growth and water status, several physiological parameters were measured in WW, WD, and RW plants. For WW plants, between 35 and 50 dps, PLA increased 6-fold (Figure 2A), leading to a rosette expansion rate of 3.7 ± 0.8 cm^2^ per day, and FW and DW increased 10.4- and 13.4-fold, respectively (Figures 2B,C). The lower increase of PLA in comparison to FW and DW may be explained by overlaying the mature leaves occurring from 46 dps, which hinders PLA measurement and results in underestimated values. FW and DW of WW plants displayed a two-phase curve characterized by a slight increase from 35 to 41 dps and a greater increase from 41 to 50 dps (Figure 2B). In WD plants, PLA decreased significantly after 10 dpwa (=45 dps), as a first mark of growth reduction on the onset of the Wi (Figure 2A). FW of WD plants was not significantly different from that of the WW plants until 8 dpwa (=43 dps) but clearly decreased afterward and had a 3.5-fold reduction at 15 dpwa (=50 dps) (Figure 2B). The DW gradually increased until 12 dpwa (=47 dps) (Figure 2C) demonstrating the accumulation of organic matter. After a period of stagnation, a loss of DW was recorded at 14 dpwa (=49 dps). For RW plants, PLA was significantly higher at 50 dps than that in WD plants (ca. 4.3-fold increase), but remained significantly different from that of the WW plants. A similar profile of water-deficit response was observed for both FW and DW.

The water absorption capacity of leaves was evaluated by measuring the TW and the RWC (Figures 2D,E). In WW plants, TW gradually increased up to 12-fold between 35 and 50 dps. RWC was stable (87.9 ± 5.8%), which indicated a good water status of WW Col-0 plants, thereby validating them as controls for studying the impact of water stress. In comparison, TW and RWC decreased significantly in WD plants from 10 dpwa (=45 dps).

In the 15 days of growth monitoring, the WC of rosettes of WW plants was stable and ranged between 92.2% and 94.4% (the mean value of 93.3 ± 1.1%) (Figure 2F). For WD plants, WC slowly decreased from 92.1 ± 0.9% at 9 dpwa (= 44 dps) to 86.2 ± 4.3% at 12 dpwa (47 dps), and this trend was further accelerated until 57.38 ± 19.97% at 15 dpwa (=50 dps). After 3 days of re-watering from 48 to 50 dps, the WC of 12-day RW plants (WC days 47 = 86.15 ± 4.29%) reached 91.9 ± 0.54% and was therefore close to that of WW plants. The same restauration of water status was also observed for RWC even if the other parameters (PLA, FW, DW, and TW) remained significantly different from those of the WW plants.

We measured SC to evaluate stomatal closing as one of the earliest mechanisms involved in the regulation of water loss. SC was stable in WW plants, with a natural variation between 160.0 ± 47.5 and 106.0 ± 46.0 mmol/m^2^/s (Figure 2G). The SC of WD plants was not significantly different from that of WW until 8 dpwa (43 dps) but then decreased rapidly to reach at 12 dpwa (47 dps) the value of 26.2 mmol/m^2^/s corresponding to an increase of stomatal closure from 75 to 92%. As of 13 dpwa (48 dps), it was no longer possible to measure SC, suggesting that stomata were completely closed. In RW plants after 3 days of re-watering, SC reached the same level as that of the WW plants at 50 dps.

**Figure 3:**
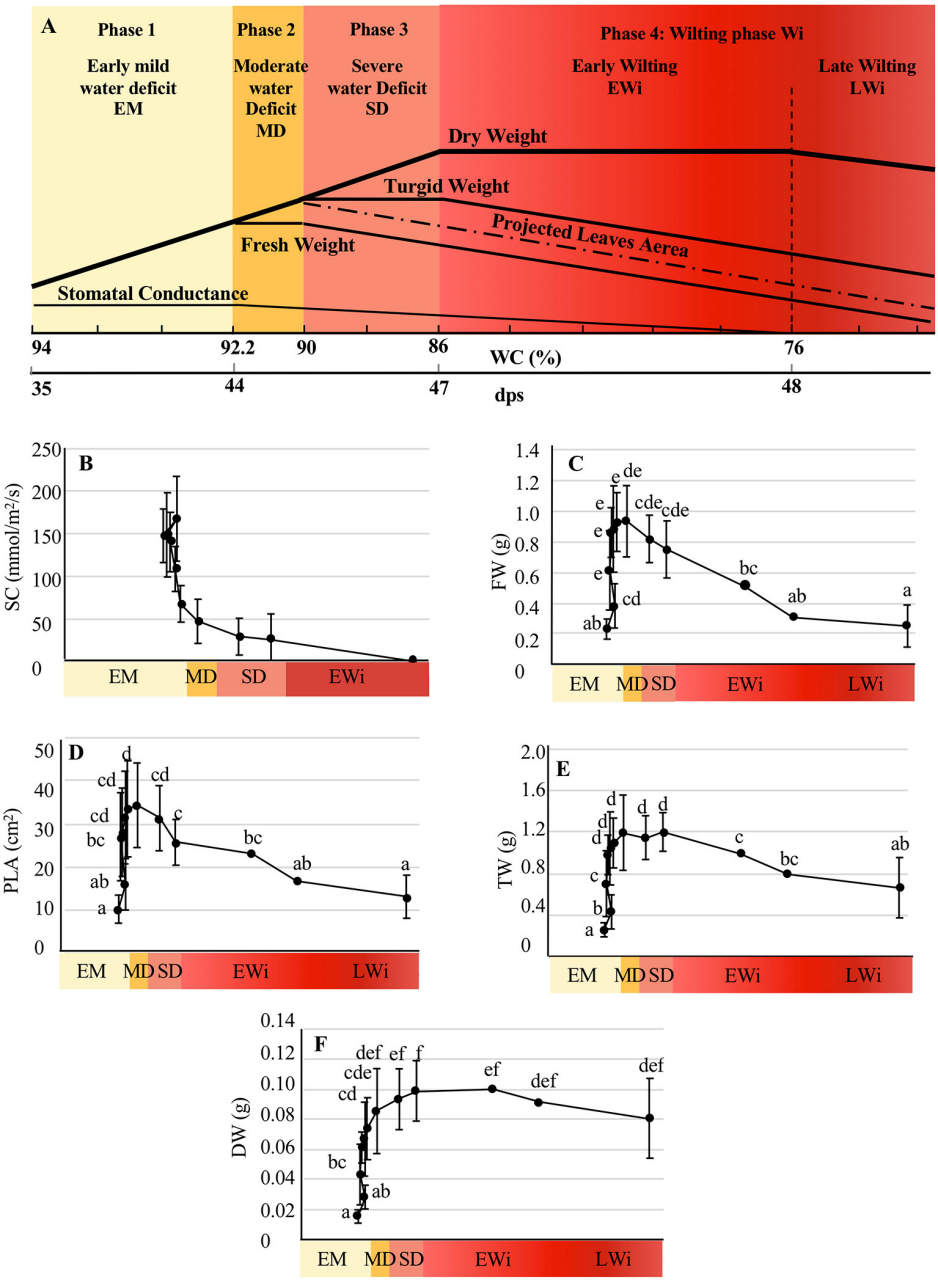
Evolution of physiological parameters under water deficit. **(A)** Determination of the four phases of water deficit as a function of WC. dps, days post-sowing. Evolution of **(B)** SC, stomatal conductance; **(C)** FW, fresh weight; **(D)** PLA, projected leaf area (PLA); **(E)** TW, turgid weight; **(F)** DW, dry weight in WD *A. thaliana* Col-0 represented as function of the water-deficit phases. The study was carried out with five plants per condition, and three independent biological repeats were performed (±SD). Statistical analysis was performed using the Kruskal–Wallis test and multiple comparisons of Dunn (*p* < 0.05) corrected by Bonferroni. Significantly different values are indicated by distinct letters.

In regard to the evolution of plant growth and physiological parameters according to the WC of WD plants, four main phases were defined (Figure 3A): (1) EM phase characterized by a WC >92.2%, an increase in FW, DW, TW, PLA, a plant growth similar to those of WW plants and a maximal SC; (2) MD phase, with a WC decreasing from 92.2 to 90%, and characterized by a drop of stomatal conduction (Figure 3B) as well as a stagnation of the FW; (3) SD phase with a WC decreasing from 90% to 86% and characterized by a decrease of FW and PLA (Figures 3C,D), and a stagnation of TW. The increase of DW was identical to that in WW plants suggesting that plants still have the capacity to synthesize organic matter; (4) Wi phase that was subdivided into early wilting (EWi) and advanced wilting (LWi). The EWi with a decrease of WC from 90 to 76% was characterized by a decrease of TW (Figure 3E) and also the stagnation of DW. The LWi is characterized by a decrease of WC below 76%, and more specifically, by a decrease of DW (Figure 3F). This late stage corresponds to plasmolysis, and probably, to some cell death (Figure 1Cf).

### Osmotic Pressure

The osmotic pressure of leaf cells was measured at 45, 47, and 49 dps by immersing leaf fragments into sucrose solutions with increasing concentrations (Table 1). From 45 to 49 dps, the osmotic pressure of WW plants was between 0.9 and 1.2 MPa. In WD plants, the osmotic pressure was higher than that of WW plants at 45 dps (=10 dpwa), corresponding to the SD phase, and further increased to 1.5–1.7 MPa at 47 dps (=12 dpwa), corresponding to the Wi phase. At 49 dps (=14 dpwa), only 33% of the tested plants showed an osmotic pressure of 2.4 MPa while massive plasmolysis and cell death were observed in the remaining 67%.

**Table 1:** Osmotic pressure (MPa) in the leaves of *Arabidopsis thaliana* Col-0 and *atesl1.02/erdl6* mutant grown under well-watered (WW) and WD culture conditions at 45, 47, and 49 days post-sowing.

**Days post-sowing**	**Osmotic pressure (MPa)**				**Water content and associated phases**
	**Well-Watered plants**		**Watered-Deprived plants**		
	**Col-0**	***atesl1.02/erdl6* mutant**	**Col-0**	***atesl1.02/erdl6* mutant**	
45	1.1–1.2	1.1–1.2	1.2–1.4	1.1–1.2	90% > WC > 86%
47	0.9–1.1	0.8–0.9	1.5–1.7	1.2–1.4	phase 3 – SD
49	1.1–1.2	0.8–0.9	2.4 (for 33% of plants)/ND	1.5–1.7	WC < 76% phase 4-LWi

*ND, not determined*.

### Content of Soluble Sugars

**Figure 4:**
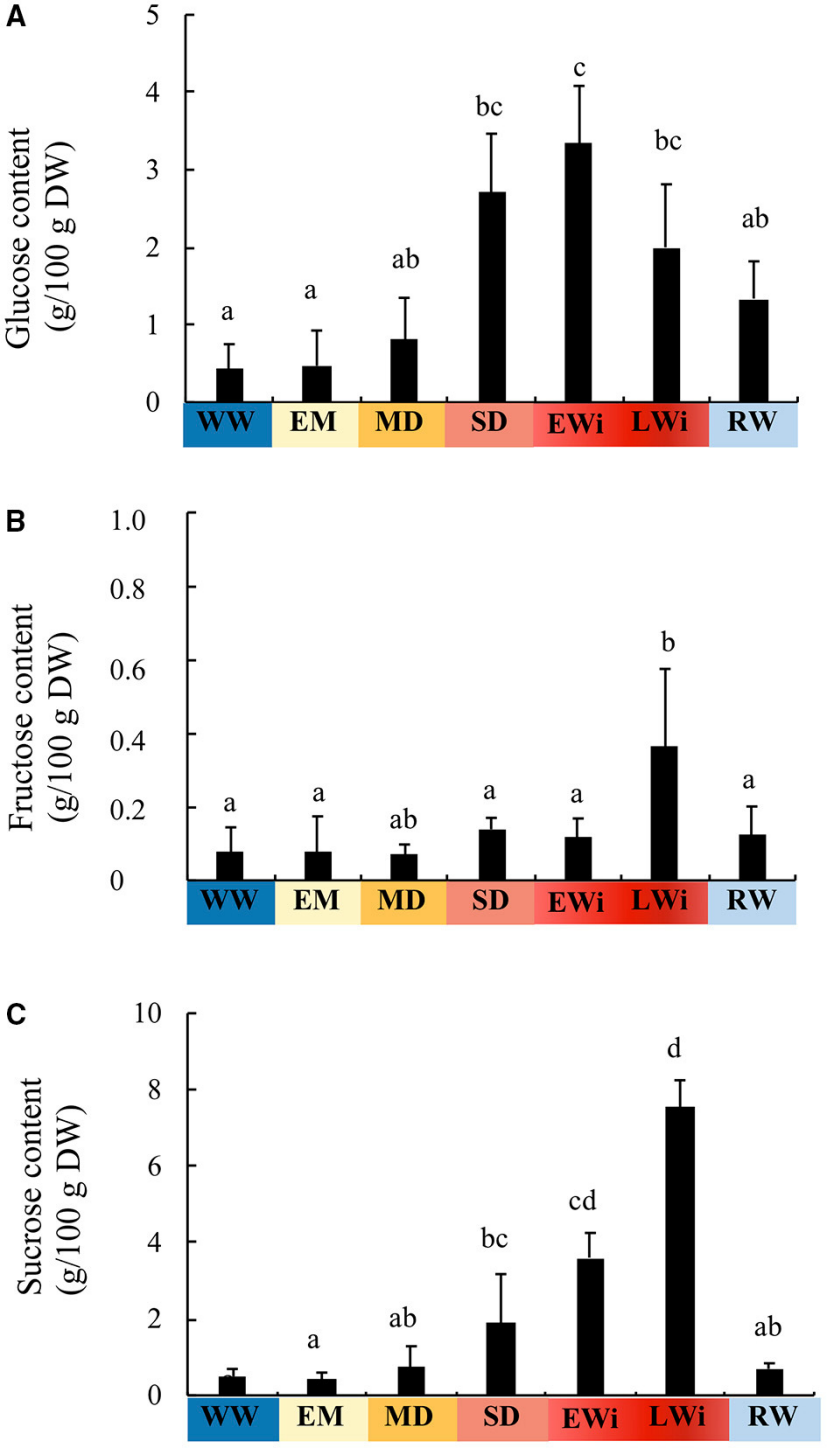
Sugar content in the leaves of *A. thaliana* Col-0. **(A)** glucose content, **(B)** fructose content, and **(C)** sucrose content as function of the four phases of water deficit: EM, early mild water deficit; MD, moderate water deficit; SD, severe water deficit; EWi, early wilting; LWi, late wilting. The study was carried out with five plants per condition and three independent biological repeats were performed (±SD). The Kruskal–Wallis test, followed by Dunn's multiple comparisons test (*p* < 0.05) with the Bonferroni correction, resulted in significantly different groups, as indicated by different letters.

As an increase in sugar content potentially play a role in the process of cell osmotic adjustment, we determined sucrose, glucose, and fructose contents in WW, WD, and RW plant rosettes, respectively (Figure 4, Supplementary Figure 1). The content of soluble sugars as a function of WC was manifested by an increase of glucose and sucrose during the transition from the MD phase (92.2% > WC > 90%) to the SD phase (90% > WC > 86%) (Figures 4A,C). The increase of fructose was delayed to the end of the Wi phase (WC < 76%) (Figure 4B). Glucose and sucrose contents in RW plants were similar to those of WD plants in MD phase (Figures 4A,C). The three sugar contents were almost restored after 3 days of rehydration.

### Expression Profiling of *AtESL* in Leaves Under Water Deficit

To highlight the role of the ESL SUTs in response to drought, the expression of 17 *AtESL* genes was measured in the leaves of plants grown under water deficiency. In the leaves of WW plants, the relative expression of 14 out of the 17 *AtESLs* was stable during growth kinetics. Only the expression of *AtESL1.02/ERDL6* gene was repressed from 49 dps (Supplementary Figure 2A), and that of two tandem genes, *AtESL3.02* and *AtESL3.03*, were induced from 49 to 50 dps, respectively (Supplementary Figures 2B,C).

**Figure 5:**
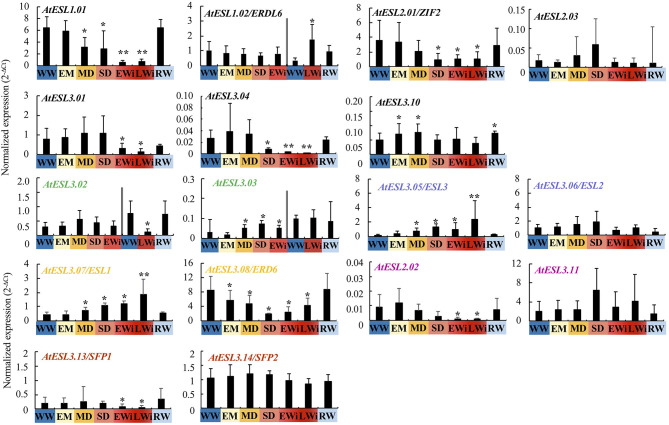
Relative expression of 17 *AtESL* in *A. thaliana* Col-0 in WW, WD, and RW plants as the function of the four phases of water deficit: EM, early mild water deficit; MD, moderate water deficit; SD, severe water deficit; EWi, early wilting; LWi, late wilting. The 2^−ΔCt^ values are normalized according to *AtPP2a* expression. On the left of the double bar, the level of gene expression does not show any significant variation during the growth kinetic under WW conditions: the gene expression in EM, MD, SD, and EWI phases is compared to the mean value measured under WW condition during these periods. On the right of the double bar, the gene expression being significantly different at the end of the growth kinetics (49–50 dps): the gene expression in LWi and RW is compared to that observed under WW conditions those late developmental phases. The study was carried out with five plants per condition and three independent biological repeats were performed (±SD). The asterisks represent the significantly different values determined by the Kruskal–Wallis test followed by Dunn's multiple comparisons test (*p < 0.05; **p < 0.001) with the Bonferroni correction.

The relative expression of 17 *AtESL* genes was presented as a function of the four phases of water deficit (Figure 5). The relative expression of *AtESL1.01* decreased from the MD phase and became minimal in the Wi phases (8-fold less than in WW plants), while that of *AtESL1.02/ERDL6*, showed a 4.8-fold increase during LWi phase. This result demonstrated that *AtESL1* was differently regulated during water deficit and *AtESL1.02/ERDL6* was specifically induced during LWi while *AtESL1.01* was reduced much earlier.

The relative expression of *AtESL2.01/ZIF2* and of *AtESL3.04* were repressed in SD and Wi phases, respectively. *AtESL3.01* was repressed only in Wi phases. In RW plants, the expression levels of these *AtESL* genes were comparable to those in WW plants. The expression of *AtESL3.10* seemed to increase during EM and MD phases in comparison to WW plants. *AtESL2.03* did not respond to water deficit.

The *ESL* copies of each of the five tandem pairs, *AtESL3.02-AtESL3.03, AtESL3.05/ESL3-AtESL3.06/ESL2, AtESL3.07/ESL1-AtESL3.08/ERD6, AtESL2.02-AtESL3.11*, and *AtESL3.13/SFP1-AtESL3.14/SFP2*, were differently regulated according to water deficit intensity (Figure 5). *AtESL3.03* was early induced from MD to EWi phase (about 2-fold induction) while *AtESL3.02* was 6-fold repressed in the LWi phase. *AtESL3.05/ESL3* and *AtESL3.07/ESL1* displayed the same expression profile in WD plants, marked by an induction starting from MD (3.7 and 1.7 times, respectively) to advanced Wi phase (11.9 and 4.4 times, respectively) compared to the WW phase. Inversely, *AtESL3.06/ESL2* (a tandem copy of *AtESL3.05/ESL3*) did not respond to water deficit, and *AtESL3.08/ERD6* (a tandem copy of *AtESL3.07/ESL1*) was repressed very early during the MD phase (1.8-fold) and showed the lowest expression level in the SD phase (4.7-fold). *AtESL2.02* and *AtESL3.13/SFP1* were repressed only in early and late Wi phases (respectively, 88 and 91% for *AtESL2.02* and 57 and 75% for *AtESL3.13/SFP1*) while *AtESL3.11* and *AtESL3.14/SFP2* were not responding to water deficit. The relative expression of all *AtESL* in RW plants (WC = 91.99 ± 0.54%) was significantly similar to that observed in WW plants, except for *AtESL3.10*.

Altogether, the previous results show that in WD plants among the 17 *AtESL* genes, 5 were not significantly affected by water deficit (*AtESL2.03, AtESL3.06/ESL2, AtESL3.10, AtESL3.11*, and *AtESL3.14/SFP2*) and 4 others were clearly induced; 3 early from the MD phase (*AtESL3.03, AtESL3.05/ESL3*, and *AtESL3.07/ESL1*) and one later during the LWi phase (*AtESL1.02/ERDL6*). Furthermore, the expression of eight genes was repressed either as early as in the EM phase (*AtESL3.08/ERD6)* or later on—in the MD (*AtESL1.01*), SD (*AtESL2.01/ERDL6, AtESL3.04)*, EWi phases (*AtESL2.02/ZIF2* and *AtESL3.01, AtESL3.13/SFP1*), or by the late Wi phase (*AtESL3.02*).

**Figure 6:**
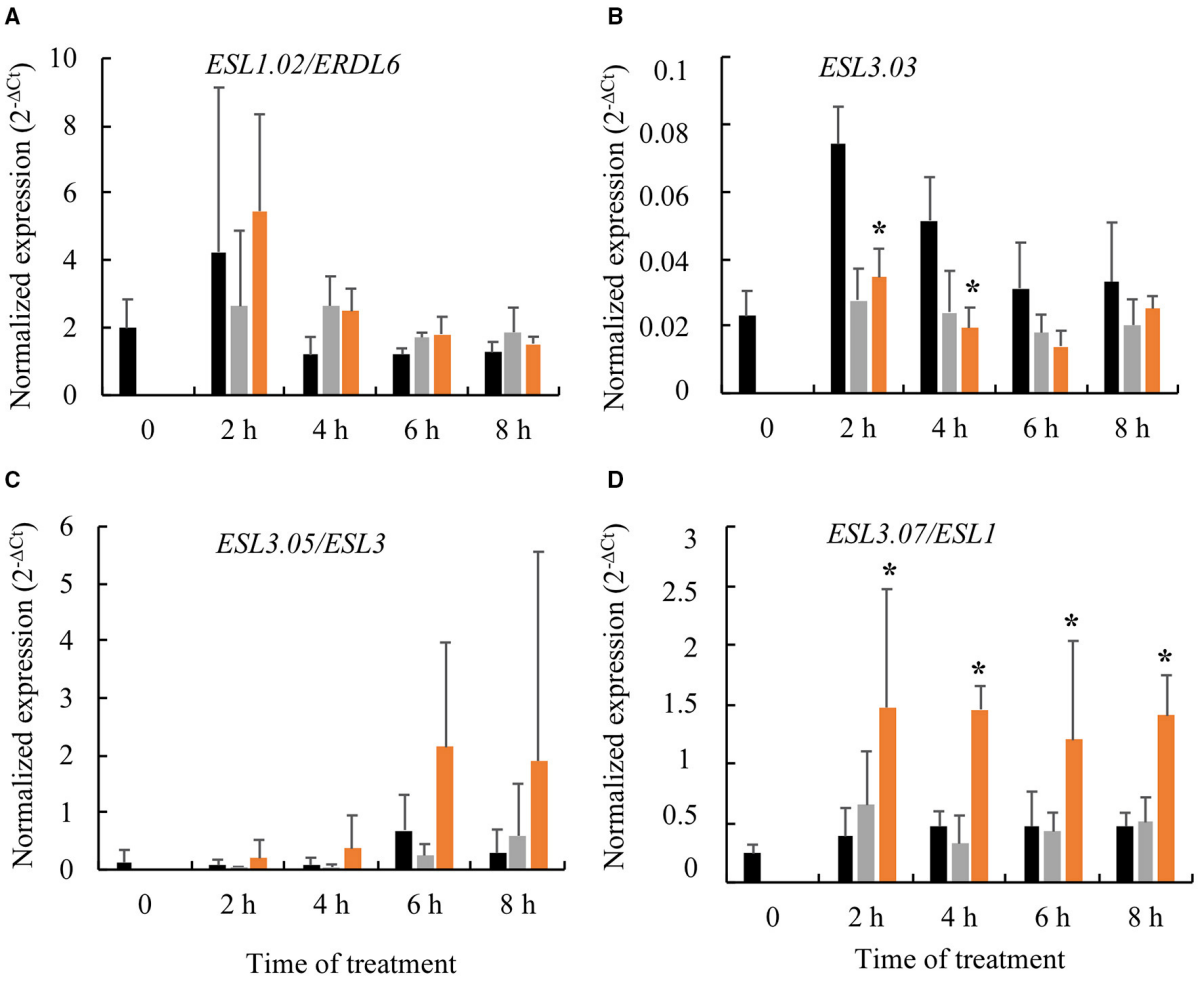
Relative expression of four *AtESL* genes in response to abscisic acid (ABA) pulverization on leaves of *A. thaliana* Col-0. Black: control, Gray: DMSO, Orange: DMSO + ABA (750 μM). **(A)** *ESL1.02/ERDL6*, **(B)** *ESL3.03*, **(C)** *ESL3.05/ESL3*, and **(D)** *ESL3.07/ESL1*. 2^−ΔCt^ values are normalized according to *AtPP2a* expression and represent the mean of three biological repeats (±SD). The asterisks represent the significantly different values determined by the Kruskal–Wallis test followed by Dunn's multiple comparisons test (*p* < 0.05) with the Bonferroni correction.

As ABA is a phytohormone well-known to be involved in drought response pathway, we analyzed the effect of this phytohormone on the expression of the four drought-induced *AtESL*. In leaves sprayed with the 750 μM ABA solution, the expression of *ESL3.07/ESL1* was significantly induced, whereas those of *ESL1.02/ERDL6, ESL3.03*, and *ESL3.05/ESL3* were not (Figure 6).

### Expression Profiling of Other Vacuolar Transporters and Invertases in Leaves Under Water Deficit

**Figure 7:**
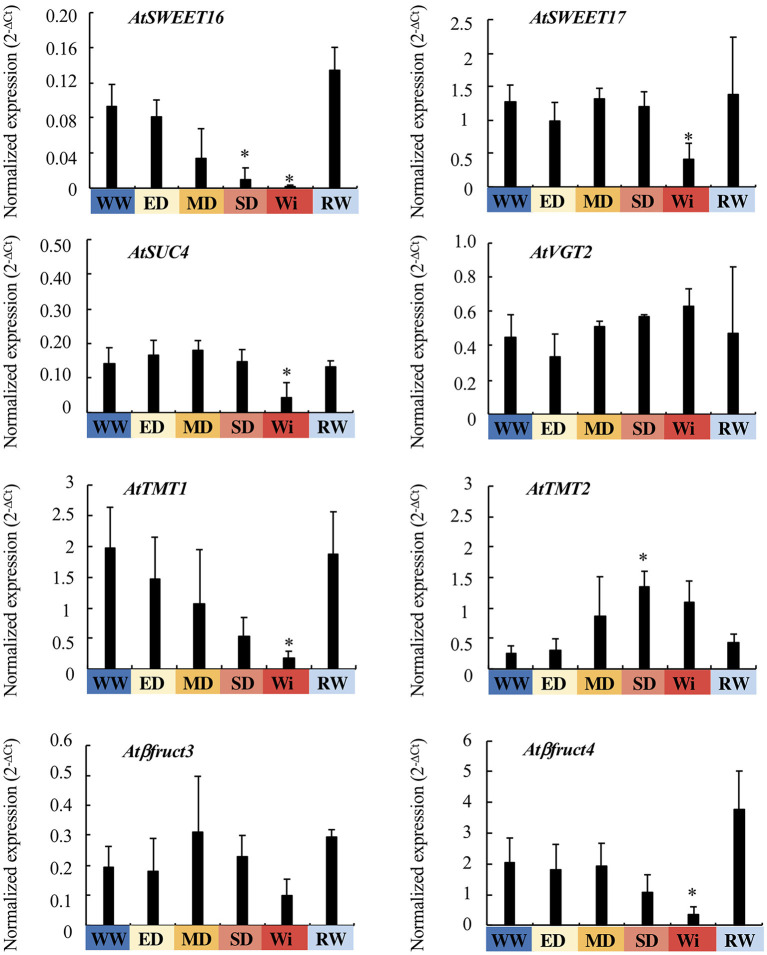
Relative expression of vacuolar sugar transporter (SUT) and invertase genes in the leaves of *A. thaliana* Col-0, under WW, WD, and RW conditions. The 2^−ΔCt^ values are normalized according to *AtPP2a* expression and are represented as the function of the four phases of water deficit: EM, early mild water deficit; MD, moderate water deficit; SD, severe water deficit; Wi, wilting phase. The value Wi represents the average values of EWi and LWi when these values are not different. The study was carried out with five plants per condition, and two independent biological repeats were performed (±SD). The asterisks correspond to significant differences compared to the WW plants, determined by the Mann–Whitney test (*p* < 0.05).

To have a global overview of the regulation of SUTs involved in sugar exchanges between the cytosol and the vacuole in response to water deficit, the relative expression of other SUT genes described to be located on the tonoplast, as well as the vacuolar invertases, was analyzed (Figure 7). *AtSWEET16* was repressed (89%) at WC of 90% during the SD phase, whereas the repression of *AtSWEET17, AtSUC4*, and *AtTMT1* occurred later on, during the Wi phase (the repression of 67, 71, and 90%, respectively). The expression of the *AtTMT2* gene was transiently induced in the SD phase (4.96-fold) in comparison to WW plants. There were no changes in *AtVGT2* expression during water deficit. For the vacuolar invertases, the expression of *At*β*fruct4* was significantly repressed (83%) during the Wi phase, whereas that of *At*β*fruct3* was not.

### Characterization of Four *atESL* Mutants

**Figure 8:**
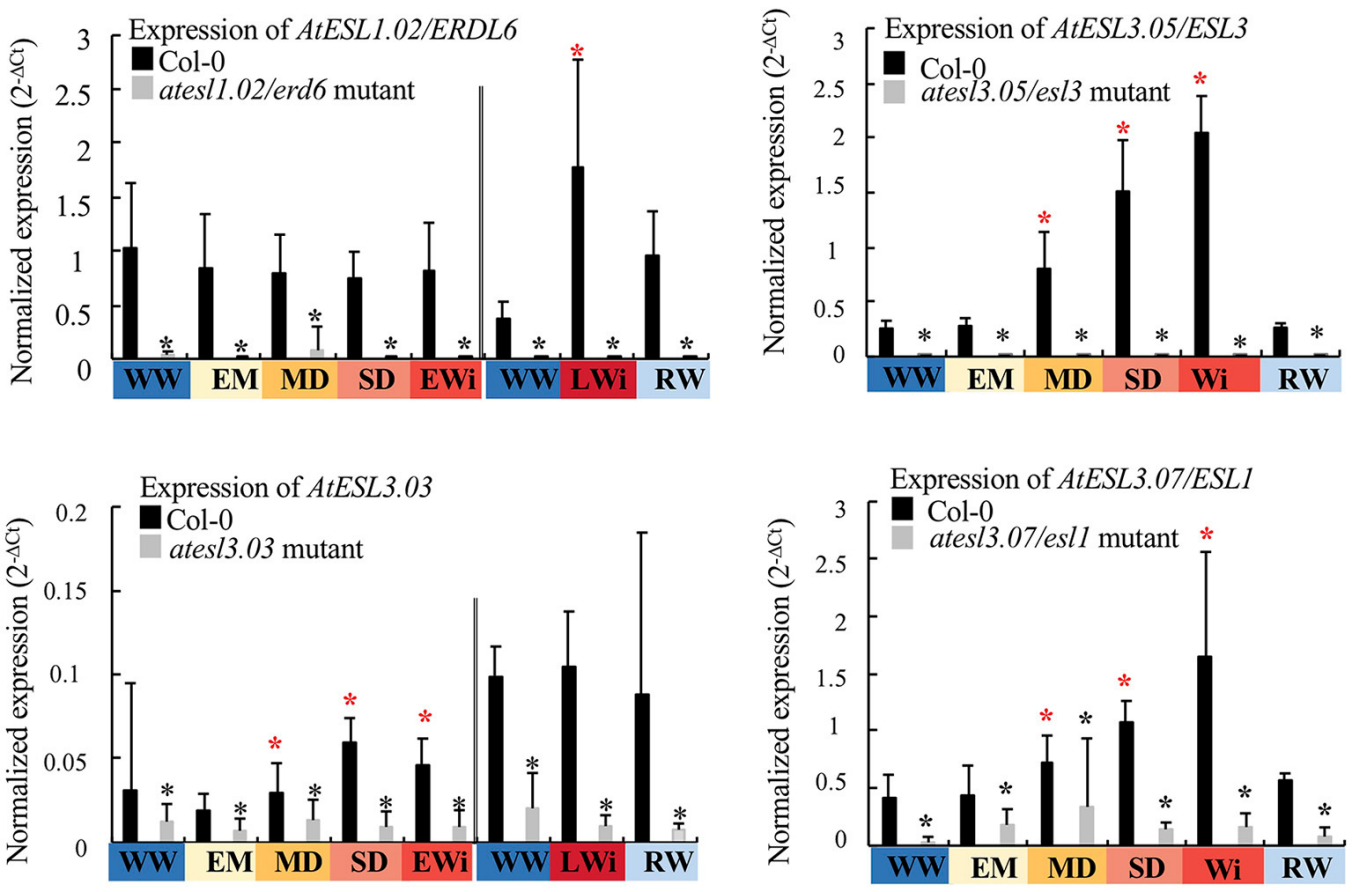
Expression level of *AtESL1.02/ERDL6, AtESL3.03, AtESL3.05/ESL3*, and *AtESL3.07/ESL1* in the leaves of T-DNA insertional mutants: *atesl1.02/erdl6, atesl3.03, atesl3.05/esl3*, and *atesl3.07/esl1*. Comparison of the expression levels of the corresponding gene in its respective mutant under WW, WD, and after RW conditions as a function of the four phases of water deficit: EM, early mild water deficit; MD, moderate water deficit; SD, severe water deficit; EWi, early wilting; LWi, late wilting; Wi, wilting phase; Wi, wilting phase, the average of the values of EWi and LWi when these values are not different. The 2^−ΔCt^ values are normalized according to *AtPP2a* expression. On the left of the double bar, the level of gene expression does not show any significant variation during the growth kinetics under WW conditions: the gene expression in EM, MD, SD, and EWI phases is compared to the mean value measured under the WW condition during these periods. On the right of the double bar, the gene expression being significantly different at the end of the growth kinetics (49–50 dps): the gene expression in LWi and RW is compared to that observed under WW conditions in those late developmental phases. Each value represents the mean of three biological repeats (±SD). Black asterisks correspond to significant differences between *atesl* mutant and Col-0 plants, determined by the Mann–Whitney test (*p* < 0.05). Red asterisks correspond to significant differences determined by the Kruskal–Wallis test, followed by a Dunn's test for multiple comparisons with Bonferroni correction (*p* < 0.05).

To highlight the role of ESLs in *Arabidopsis* response to water deprivation, T-DNA insertional mutants were identified for each of the four *AtESL* genes that were clearly induced under water deficit (*AtESL1.02/ERDL6, AtESL3.03, AtESL3.05/ESL3*, and *AtESL3.07/ESL1*). When compared to Col-0 genotype, *atesl1.02, atesl3.03, atesl3.05*, and *atesl3.07* showed the repression of the disrupted gene (98, 98, 87, and 55%, respectively). Furthermore, in each mutant, any upregulation of the corresponding mutated gene was observed in response to water depletion indicating that the mutants were suitable to study the response to water deficit (Figure 8 and Supplementary Figure 3A).

**Figure 9:**
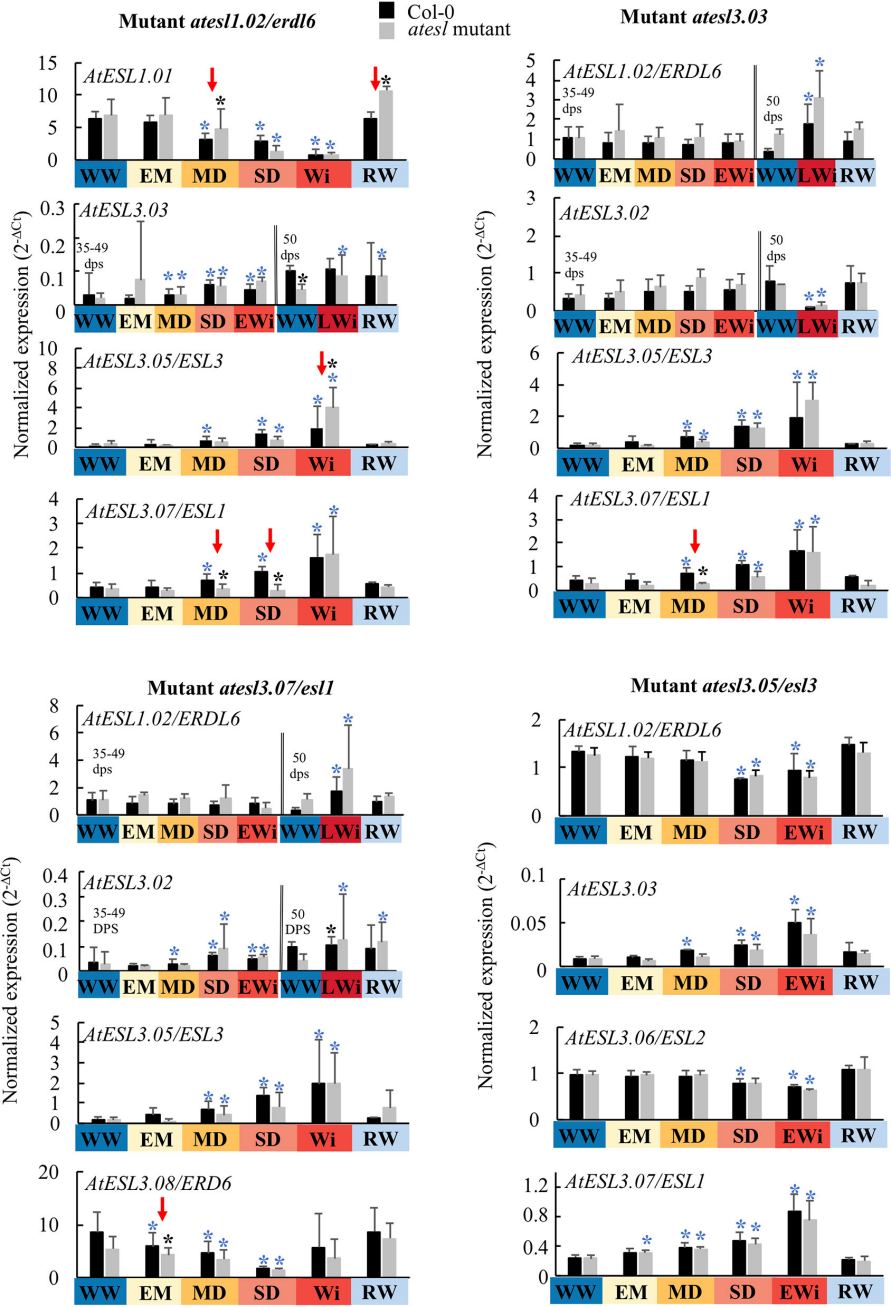
Expression level of *AtESL* in the *atesl* mutants (*atest1.02/erdl6, atesl3.03, atesl3.05/esl3*, and *atesl3.07/esl1*) in WW, WD, and RW plants compared to Col-0 plants as a function of the four phases of water deficit: EM, early mild water deficit; MD, moderate water deficit; SD, severe water deficit; EWi, early wilting; LWi, late wilting; Wi, wilting phase, the average of the values of EWi and LWi when these values are not different. On the left of the double bar, the level of gene expression does not show any significant variation during the growth kinetics under WW conditions: the gene expression in EM, MD, SD, and EWI phases is compared to the mean value measured under the WW condition during these periods. On the right of the double bar, the gene expression being significantly different at the end of the growth kinetics (49–50 dps): the gene expression in LWi and RW is compared to that observed under WW conditions in those late developmental phases. The study was carried out with five plants per condition and three independent biological repeats were performed (±SD). Black asterisks correspond to significant differences between *atesl* mutant and Col-0 plants, determined by the Mann–Whitney test (*p* < 0.05). Blue asterisks correspond to significant differences between WD or RW in comparison to the WW condition determined using the Kruskal–Wallis test followed by Dunn's multiple comparisons test (*p* < 0.05).

Expression profiles of the four induced *AtESLs* (*AtESL1.02/ERDL6, AtESL3.03, AtESL3.05/ESL3*, and *AtESL3.07/ESL1*), their tandem copies (*AtESL3.02, AtESL3.06/ESL2*, and *AtESL3.08/ERD6*), and *AtESL1.01* were determined in each single mutant under water deficit and after re-watering (Figure 9). In the *atesl1.02/erdl6* mutant, the reduction of the expression of *AtESL1.01* gene was delayed to the SD phase when compared to the wild Col-0 genotype for which its repression started at the MD phase. *AtESL1.01* gene expression manifested a 5-fold decrease in the SD phase and a further 8-fold repression during the EW phase. In RW plants, *AtESL1.01* expression was restored and reached higher levels than that of the Col-0 genotype. For the same mutant, *AtESL3.05* gene was more strongly expressed during the EWi step than in Col-0. The increase of *AtESL3.07* expression was delayed to the EW phase in comparison to wild-type genotype, for which the induction started to the MD phase. In the *atesl3.03* mutant, the induction of *AtESL3.07* expression took place later than in Col-0 in the SD phase instead of the MD phase. In *atesl3.07/esl1* mutant, the reduction of the expression of its tandem gene *AtESL3.08/ERD6* was emphasized during the EM phase. In the *atesl3.05/esl3* mutant, no significant differences in the expression of *AtESL* genes were found when compared to those of the wild genotype.

**Figure 10:**
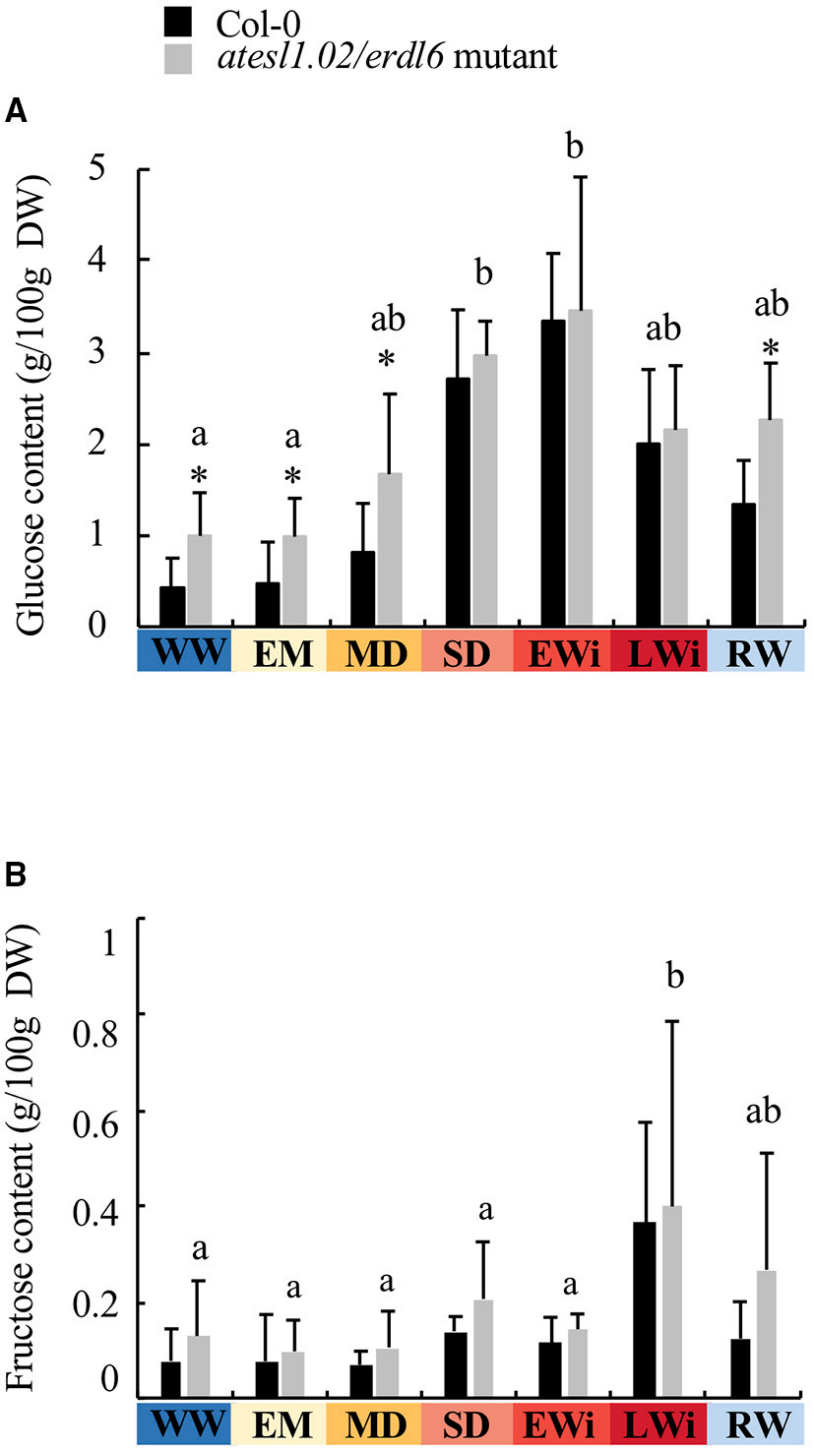
**(A)** Glucose and **(B)** fructose contents in the leaves of *A. thaliana* Col-0 and *atesl1.02/erdl6* mutant in WW, WD, and RW plants as a function of the four phases of water deficit: EM, early mild water deficit; MD, moderate water deficit; SD, severe water deficit; EWi, early wilting; LWi, late wilting. The values represent the mean of three biological replicates (±SD). Letters indicate statistically different values between the water-deficit phases determined by the Kruskal–Wallis test (*p* < 0.05) followed by Dunn's multiple comparisons test. Black asterisks correspond to significant differences between *atesl1.02/erdl6* mutant and Col-0 plants, determined by the Mann–Whitney test (*p* < 0.05).

To determine whether the content of soluble sugars was affected in the mutant lines, glucose, fructose, and sucrose contents were measured in the leaves of WW, WD, and RW plants. No differences in sugar content could be detected in *atesl3.03, atesl3.05/esl3*, and *atesl3.07/esl1* mutants as well as for fructose (data not shown) and sucrose content in the mutant *atesl1.02/erdl6* (Supplementary Figure 4). Inversely, the glucose content was strongly increased in *atesl1.02*/*erdl6* mutant in WW and WD plants during EM and MD phases, as well as in RW plants (Figure 10, Supplementary Figure 3B). In the mutant *atesl1.02/erdl6*, the osmotic pressure in WW plants seemed to be slightly lower than that of Col-0. In WD plants, the osmotic pressure of the mutant *atesl1.02/erdl6* increased later (at 47 dps corresponding to the SD phase; Table 1) compared to Col-0, and is lower to the osmotic pressure of Col-0 at 49 dps. In the other three mutants (*atesl3.03, atesl3.07/esl1*, and *atesl3.05/esl3*), no significant differences with Col-0 genotype were observed in terms of osmotic pressure and soluble sugars. Vegetative growth (PLA, FW, TW, and DW) and physiological parameters (WC, RWC, and SC) of the four mutants *atesl1.02/erdl6, atesl3.03, atesl3.05/esl3*, and *atesl3.07/esl1* did not show any significant differences in respect to those of the wild genotype under the conditions of WW, WD, and RW (Supplementary Figure 5).

## Discussion

**Figure 11:**
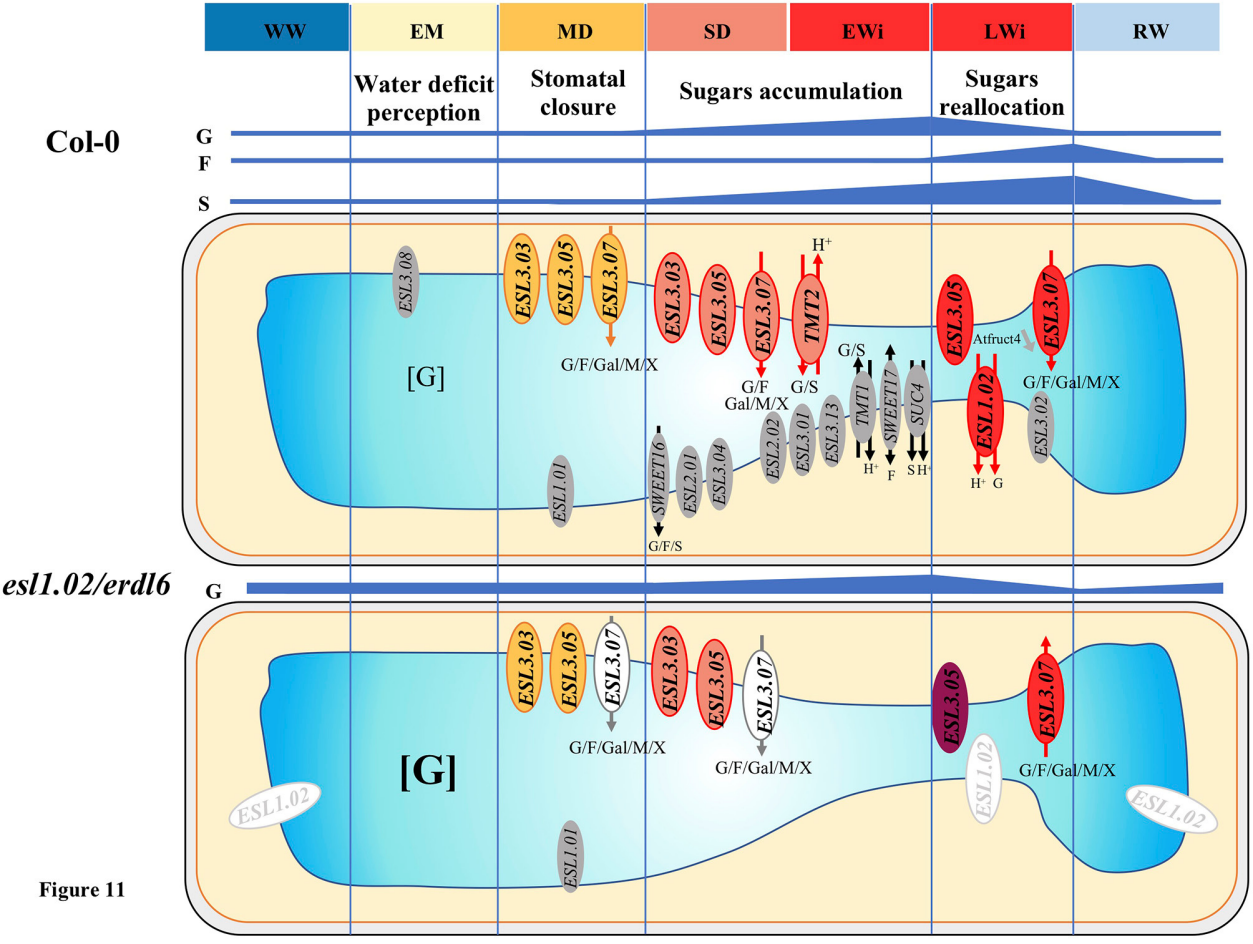
Summary of the evolution of the sugar content and of the expression of vacuolar SUTs in the leaves of *A. thaliana* Col-0 and *esl1.02/erdl6* mutant, under water deficit. WW means well-watered condition (control); water deficit is split into different phases (EM, early mild water deficit; MD, moderate water deficit; SD, severe water deficit; EWi, early wilting; LWi, late wilting). RW means re-watering. G, F, S, Gal, M, and X stand for glucose, fructose, sucrose, galactose, mannose, and xylose. Gray color indicates repressed transporters; orange, light, and dark red indicate different levels of induction. In *esl1.02/erdl6*, the induction of *ESL3.07* under water deficit is delayed from MD to LWi phases and *ESL3.05* is more strongly induced in the LWi phase in comparison to Col-0.

The present study aims to correlate the expression of *AtESL* SUTs in the response of *A. thaliana* to water deficit intensity, in terms of growth, physiological, and leaf sugar metabolic behavior, to identify their role in plant response to water deficit. Using 35-day-old *Arabidopsis* plants and inducing water stress by withholding water, we were able to define four different phases in the stress period and analyzed, for each phase, the regulation of *AtESL* transporter genes (Figure 11).

In the EM phase (WC ranged between 98 and 92.2%), the physiological parameters and sugar contents for WD plants did not display any significant differences compared to those of WW plants. Thus, the progressive leaf water loss (5.8%) has not yet affected the plant phenotype (Figures 1–3). However, *AtESL3.08/ERD6* gene repression corresponded to the onset of EM phase and was maintained all along the water-deficit period. Therefore, this *ESL* may be considered as the earliest responsive gene, which might be regulated by initial changes in water potential before the appearance of any changes in growth and physiological parameters. However, even though *AtESL3.08*/*ERD6* has been described not only as the first identified *ESL* but also as an early drought responsive gene, our results disagree with these previous reports in the fact that the expression of *AtESL3.08*/*ERD6* was described to be induced after few hours of dehydration (Kiyosue et al., 1998; Seki et al., 2002; Yamada et al., 2010). This discrepancy might be explained by the fact that in those previous studies water deficit was performed *via* dehydration instead of water withholding as in our experiments. Furthermore, we were able to show that, in WW soil cultured plants, *AtESL3.08/ERD6* was the highest expressing ESL in *Arabidopsis* organs, such as leaves, roots, buds, flowers, and siliques and that its strongest expression level is observed in roots (Slawinski et al., 2021). Using *ERD6*pro:*GUS* and *ERD6*pro:*TM-GFP* transgenic plants, Yamada et al. (2010) have demonstrated a preferential expression of *ESL3.08/ERD6* in epidermal and cortex cells. *ESL3.08/ERD6* root localization suggests that its rapid repression may be a part or a consequence of the early drought perception in roots.

In the MD phase, WC decreased from 92.2 to 90% (i.e., a net 2.2% water loss), thereby reaching a cumulative water loss of 8%, which caused stomatal closure as an early plant response to maintain the plant water state under reduced soil water potential (Costa França et al., 2000; Flexas et al., 2004; Blouin et al., 2007; Harb et al., 2010; Arve et al., 2011). ABA is known as the major plant hormone with a double function involved not only in stomatal closure, but also in the prevention of stomatal opening (Liu et al., 2005; Seki et al., 2007; Arve et al., 2011; Živanović et al., 2020). The steps that occur between the sensing of drought and the production of ABA are complex vary among plant species and are still not completely understood. Various signals, such as hydraulic pressure, ROS/Ca^2+^ waves, and peptides, contribute to this response to drought (Takahashi et al., 2020). In *Arabidopsis*, the peptide CLAVATA3/EMBRYO-SURROUNDING REGION-RELATED25 (CLE25) showed to function as a long-distance signal from roots to shoots, and causes ABA production in leaves (Christmann and Grill, 2018). In our experiments, the MD phase was associated with the induction of three SUT genes *ESL3.03, ESL3.05/ESL3*, and *ESL3.07/ESL1*, with an enhancement of the repression of *ESL1.01* and the maintained repression of *ESL3.08/ERD6*. These events marked also the onset of glucose and sucrose accumulation in leaves. Our results imply that the three induced *ESLs* (*ESL3.03, ESL3.05/ESL3*, and *ESL 3.07/ESL1*) might be involved in the accumulation of sugars. Furthermore, we were able to show that among the three induced genes only *ESL3.07/ESL1* was clearly induced when 750 μM ABA was sprayed on leaves (Figure 6). A similar induction was also observed in leaves and roots, 5 h after 100 μM ABA treatment (Yamada et al., 2010). This suggests that, in response to water deficit, the induction of *ESL3.07/ESL1* in leaves might involve an ABA-dependent pathway, whereas the induction of *ESL3.03* and of *ESL3.05/ESL3* implies an ABA-independent pathway. It is interesting to note that in the MD phase, the expression of *ESL3.07/ESL1* and *ESL3.08/ERD6* were evolving in opposite ways. In fact, the gene expression data analysis from Genevestigator databases showed that *ESL3.07/ESL1* is highly induced by ABA treatment, whereas *ESL3.08/ERD6* is strongly repressed. According to these data, Yamada et al. (2010) have shown that the expression of *ESL3.08/ERD6* is reduced in leaves and roots after ABA treatment. These results corroborate the idea of a differential regulation by ABA and water deficit of these two tandem duplicated genes and highlight the possible mechanisms of sub-functionalization.

In the SD phase (WC from 90 to 86%), although FW and PLA decreased, TW stayed stable and DW still increased as in WW plants (Figure 2). The osmotic pressure (Table 1) rose in parallel with a strong increase of glucose and sucrose content (Figure 4). This suggests that plants, which do not present Wi phenotype, still have the capacity to maintain basic metabolism despite the closure of stomata. Our microscopy observations confirmed that the cells were not only viable but also remained turgid until 47 dps (Figure 1C). Thus, in the SD phase, an important increase in glucose and sucrose amount contributes to an osmotic adjustment of cells to maintain turgid and metabolically active cells (Morgan, 1984; Hare et al., 1998; Taji et al., 2002; Moore et al., 2008; Hummel et al., 2010; Blum, 2017). Leaf expansion is certainly affected by the arrest of cell growth under enhanced dehydration, leading to the drop of cell water potential and the loss of turgidity (Hsiao, 2003; Simonneau et al., 2017; Turner, 2018). Taken together our results agree with the already established concept that the arrest of cell growth responds to a milder water deficit than that required for a photosynthetic rate reduction (dos Santos Gouvêa and Marenco, 2018; Zhao et al., 2020). Consequently, the limitation of carbon assimilation is not the real cause of cell growth arrest. The latter is mainly related to the cell choice to invest metabolites and energy for its osmotic adjustment, the signaling of reduced water potential, and cell wall stiffening to promote plant acclimation. In this context, Mewis et al. (2012) reported that drought stress enriched the nutritional quality of phloem sap in *A. thaliana* plants with 93–94% WC by increasing the amounts of free amino acids and sugars, especially sucrose. In the SD phase, the expression levels of *ESL3.03, ESL3.05/ESL3*, and *ESL 3.07/ESL1* were higher than those in the MD phase, and the expression of *TMT2* was significantly increased. On the contrary, the expression of *ESL1.01* and *SWEET16* were reduced, and the expression levels of *ESL3.08/ERD6, ESL2.01/ZIF2*, and *ESL3.04* were minimal. This confirms that *ESL3.03, ESL3.05/ESL3*, and *ESL 3.07/ESL1* are involved together with *TMT2* in sugar accumulation for an osmotic adjustment.

In the beginning of the Wi phase (WC ranged between 86 and 76%), all measured physiological parameters decreased except DW, which remained stable. The amount of sucrose in the Wi phase was doubled compared to that in the SD phase and reached a maximum. During this EWi phase, re-watering was allowed to restore all physiological parameters at least to the levels observed in the MD phase. The repression of *ESL1.01, ESL2.01/ZIF2, ESL3.04, ESL3.08/ERD6*, and *SWEET16* genes in the SD phase was maintained at a lower level. In parallel, the expression of six other SUTs (*ESL2.02, ESL3.01, ESL3.13/SFP1, TMT1, SWEET17*, and *SUC4*) was decreased. This suggests that sugar transport is highly affected. For a WC lower than 76%, all measured growth and physiological parameters, including DW, dropped down. The WD plants at the LWi step persisted its ability to absorb water even at a WC below 76%. Below this threshold, massive cell plasmolysis was observed in leaf sections and Wi became irreversible for some leaves (Figures 1B,C). Glucose content was still maintained (although a little bit decreased), whereas sucrose content increased 2-fold in comparison to that in SD phase and fructose significantly increased. As the accumulation of soluble sugars has been shown to increase during senescence in several plant species, including *Arabidopsis* (Quirino et al., 2001; Stessman et al., 2002; Diaz et al., 2005) and that sugars are considered as a metabolic signal to trigger senescence (Pourtau et al., 2006; Rolland et al., 2006), it is tempting to suggest that the wilted plants having a WC lower than 76% were going to enter the senescence stage. However, this might not be really the case as we observed the repression of *ESL3.13/SFP1*, which has been described to be specifically induced during senescence (Quirino et al., 2001). In the LWi phase, the expression of *ESL3.02* and of the vacuolar invertase *At*β*fruct4* was strongly repressed. Remarkably, among the studied genes, *AtESL1.02/ERDL6* was the only one to be specifically induced under SD. This ESL has been described to be a vacuolar proton-driven glucose exporter (Klemens et al., 2013). This gene expression is upregulated when the changes in cell status require rapid energy mobilization through the export of vacuolar sugar reserves (a transition from light to obscurity, a temperature shift from 23 to 37°C and after injury). Inversely, *AtESL1.02/ERDL6* expression is downregulated under the conditions favorable for sugar accumulation into the vacuole, such as cold stress and a high concentration of extracellular sugars (Poschet et al., 2011). These data and the characterization of *AtESL1.02/ERDL6* as a H^+^/glucose symporter provide evidence for the correlation between *AtESL1.0/ERDL6* expression and the sugar status of the cell (Poschet et al., 2011; Klemens et al., 2013). Under strong water deficit, *AtESL1.02/ERDL6* may be involved, in glucose efflux from the vacuole to cytosol, which is required for the remobilization of sugars from mature wilted leaves and reallocation to young sink organs.

Taken together, our results demonstrate that among the 17 studied *AtESL* genes, 12 respond to water deficit, 4 with an increased expression and 8 with a decreased expression. Another important result concerns the fact that tandem duplicated *ESL* copies did not share the same expression pattern dependent on the plant water status. *AtESL2.02* showed a reduced expression at WC < 86% while *ESL3.11* was insensitive. *AtESL3.02* also showed a reduced expression at WC < 76%, while *AtESL3.03* increased as WC was lowered from 92.2 to 90%. *AtESL3.05/ESL3* expression increased in the MD phase (92.2% > WC > 90%), while *AtESL3.06/ESL2* seemed to be insensitive to water depletion. *AtESL3.07/ESL1* was upregulated for WC comprised between 92.2 and 90%, whereas *AtESL3.08* was downregulated from the EM phase. *AtESL3.13/SFP1* was repressed at WC < 86%, but *AtESL3.14/SFP2* was not regulated by water depletion. These differential responses of tandem duplicated *ESL* genes provide arguments in favor of a high diversification of *AtESL* roles in the response of plants to water deficit.

To elucidate the roles of the four upregulated *AtESL* genes using water deficit, T-DNA insertional mutants were identified and characterized under normal growth conditions and water deficit. However, none of the single *atesl* mutants displayed a specific phenotype under water deficit in comparison to wild-type mutants (Supplementary Figure 2). These results were predictable as the ESL transporters belong to a multigenic subfamily. Moreover, the tonoplast location of other SUTs (SWEETs, SUCs/SUTs, TMTs, and VGT) implies possible compensatory effect(s) between them.

In WW *atesl1.02/erdl6* mutant plants, the repression of *AtESL1.02/ERDL6* gene resulted in higher leaf glucose content than that in the wild genotype. This difference was observed in WD plants, until the MD phase, and in RW plants. This increase of glucose in *atesl1.02/erdl6* mutant happens due to glucose accumulation into the vacuole as *AtESL1.02/ERDL6* mutation avoids glucose efflux toward the cytosol (Poschet et al., 2011). In SD and Wi phases, no significant differences in the glucose content could be detected between *atesl1.02/erdl6* mutant and Col-0. This indicates that even though the mutant *atesl1.02/erdl6* presented higher glucose content in leaves under SD and EWi, it was still able to accumulate glucose involving an AtESL1.02/ERDL6 transport activity. Glucose accumulation in these two phases was certainly due to the activity of ESL3.03, ESL3.05/ESL3, and ESL3.07/ESL1. However, it is worth to note that the expression of *AtESL3.07/ESL1* was upregulated from the beginning of the MD phase in the wild genotype, while in *atesl1.02/erdl6* mutant its induction was delayed at the Wi phase (WC < 86%). These results suggest that in the mutant *atesl1.02/erdl6* the upregulation of *AtESL3.07/ESL1* expression may be inhibited by high glucose content in MD and SD phases. In Wi *esl1.02/erdl6* plants, the evolution of glucose content is similar to that measured in Col-0, which implies the involvement of other SUTs for glucose export from the vacuole. The slightly induced expression of *AtESL3.05/ESL3* in comparison to Col-0 plants suggests that this gene might compensate the repression of *AtESL1.02/ERDL6. One* can also imagine that in response to SD *ESL3.07/ESL1*, which is induced at this stage similar to the level in the wild type, could probably be involved in hexose remobilization into the cytosol. As AtESL3.07/ESL1 has been characterized as a low-affinity facilitator for hexose transport (Yamada et al., 2010), our hypothesis implies that the aforementioned transporters could exert a bidirectional control of glucose fluxes between the vacuole and cytosol.

## Conclusion

In conclusion, *Arabidopsis* and other plants respond in a similar way to water deficit with a decrease of WC, SC, FW, PLA, and DW and a concomitant increase of sugars and other compatible osmolytes (Mewis et al., 2012; Sperdouli and Moustakas, 2012; dos Santos Gouvêa and Marenco, 2018; Zhao et al., 2020). Although more than 1,000 genes have been identified to be involved in drought response (Seki et al., 2002; Shinozaki and Yamaguchi-Shinozaki, 2007; Lawlor, 2013; Fang and Xiong, 2015), only a few of them have been characterized in terms of induced tolerance to water depletion, combined with enhanced plant productivity in model plants, as well as in important agricultural crops (Skirycz et al., 2011). In *A. thaliana*, the most studied genes, whose expression was regulated in response to moderate water stress, are dehydrins, LEA, aquaporins, K^+^ ionic channels, and ESL SUTs. Water depletion responsiveness of *ESL* genes and their putative localization on the tonoplast, already demonstrated for a few of them, assumed the functional importance of these transporters through their involvement in subcellular sugar partitioning. In our study, the profiling of 17 *Arabidopsis ESL* genes revealed that 12 are responsive to water deficit, 4 upregulated, and 8 downregulated. These 12 *AtESLs* genes were differentially expressed depending on the physiologically defined phases of the plant water status. The comparison of the four *atesl* single mutants and the wild-type Col-0 under well-watering and water-deprivation growth conditions did not reveal any phenotypic difference in response to water deficit. The latter is probably due to the functional redundancy of ESLs and their synergistic actions; therefore, it will be helpful to characterize multiple mutants for ESL and/or other tonoplastic SUTs. The differential expression of each of the tandem duplicated *AtESL* genes in response to water stress is in favor of their plausible functional diversity. Our results corroborate the hypothesis of the acquisition of new physiological functions by the ESLs, which favors plant plasticity to cope up with environmental constraints.

## Data Availability Statement

The original contributions presented in the study are included in the article/Supplementary Material, further inquiries can be directed to the corresponding author/s.

## Author Contributions

AI, FD, LS, ML, and RA designed the experiments, discussed the data, and wrote the article. AI, CA, FD, FT, LS, ML, and RA performed the experiments. All authors approved the published version of the manuscript.

## Conflict of Interest

The authors declare that the research was conducted in the absence of any commercial or financial relationships that could be construed as a potential conflict of interest.

## Publisher's Note

All claims expressed in this article are solely those of the authors and do not necessarily represent those of their affiliated organizations, or those of the publisher, the editors and the reviewers. Any product that may be evaluated in this article, or claim that may be made by its manufacturer, is not guaranteed or endorsed by the publisher.

## Supplementary Material

Supplementary Figure 1 Sugar content in the leaves of *Arabidopsis thaliana* Col-0. **(A)** Total sugar content, **(B)** glucose content, **(C)** fructose content, and **(D)** sucrose content at different days post-sowing. Well-watered (WW) (circle), water-deprived (WD; triangle), and re-watered (RW; square). The study was carried out with five plants per condition, and three independent biological repeats were performed (±SD). Dps, days post-sowing. The blue asterisks correspond to significant differences compared to the WW plants, determined by the Mann–Whitney test (*p* < 0.05). The black asterisks correspond to significant differences determined by the Kruskal–Wallis test, followed by Dunn's multiple comparisons test (^*^*p* < 0.05; ^**^ *p* < 0.001).

Supplementary Figure 2 Leaf relative expression of **(A)** *AtESL1.02/ERDL6*, **(B)** *AtESL3.02*, and **(C)** *AtESL3.03* during the development of *A. thaliana* Col-0 under WW condition. 2^−ΔCt^ values are normalized according to *AtPP2a* expression. The study was carried out on five plants and in three independent biological replicates. The asterisks represent the significantly different values determined by the Kruskal–Wallis test, followed by Dunn's multiple comparisons test (*p* < 0.05) with the Bonferroni correction.

Supplementary Figure 3 **(A)** Expression level of *AtESL1.02/ERDL6, AtESL3.03, AtESL3.05/ESL3*, and *AtESL3.07/ESL1* in the leaf of T-DNA insertional mutants: *atesl1.02/erdl6, atesl3.03, atesl3.05/esl3*, and *atesl3.07/esl1*. Comparison of the expression levels of the corresponding gene in its respective mutant and in the Col-0 during the growth kinetics in WW plants. The 2^−ΔCt^ values are normalized according to *AtPP2a* expression. Black asterisks correspond to significant differences between *atesl* mutant and Col-0 plants, determined by the Mann–Whitney test (*p* < 0.05). Red asterisks correspond to significant differences determined by the Kruskal–Wallis test, followed by a Dunn's test for multiple comparisons with the Bonferroni correction (*p* < 0.05). **(B)** Glucose and fructose contents in the leaf of *A. thaliana* Col-0 and *atesl1.02/erdl6* mutant during the growth kinetics in WW plants and after RW plants. Each value represents the mean of three biological repeats (±SD). Blue asterisk correspond to statistically different values along the kinetics, determined by the Kruskal–Wallis test (*p* < 0.05) followed by the Dunn's multi-comparison test. Black asterisks correspond to significant differences between *atesl1.02/erdl6* mutant and Col-0 plants, determined by the Mann–Whitney test (*p* < 0.05).

Supplementary Figure 4 Glucose and sucrose content in leaves of *A. thaliana* of Col-0 and the *atesl* mutants (*1.02/erdl6, atesl3.03, atesl3.05/esl3*, and *atesl3.07/esl1*) grown under WW, WD, and RW plants. Sugar contents are presented as a function of the four water-deficit phases defined according to leaf water content (WC%). WW, well-watered; EM, early mild water deficit; MD, moderate water deficit; SD, severe water deficit; EWi, early wilting; LWi, late wilting. Statistical analysis was performed using the Kruskal–Wallis (*p* < 0.05) test and Mann–Whitney pairwise comparison test (*p* < 0.05). Significantly different values are indicated by distinct letters and by an asterisk, respectively.

Supplementary Figure 5 Characterization of the effect of water deficiency on the *Arabidopsis A. thaliana* Col-0 and the *atesl* mutants (*1.02/erdl6, atesl3.03, atesl3.05/esl3*, and *atesl3.07/esl1*) grown under WW, WD, and RW plants. **(A: a)** PLA: projected leaf area, **(B: b)** FW: fresh weight, **(C:c)** DW: dry weight, **(D: d)** TW: turgid weight, **(E: e)** RWC: relative water content, **(F: f)** WC: water content, **(G: g)** SC: stomatal conductance. Col-0 (circle), *atesl1.02/erdl6* (triangle), *atesl3.03* (diamond), *atesl3.07/esl1* (square), and *atesl3.05/esl1* (cross). The study was carried out with five plants per condition, and three independent biological repeats were performed (±SD). Statistical analysis was performed using the Mann–Whitney pairwise comparison test (*p* < 0.05). Significantly different values are indicated by asterisk.

Supplementary Table 1 Primers used in quantitative PCR (qPCR) for gene expression analysis and used for genotyping.
