# Supplementary figures and images for: Responsiveness of Early Response to Dehydration Six-Like Transporter Genes to Water Deficit in Arabidopsis thaliana Leaves
Source: Front Plant Sci. 2021 Aug 16;12:708876. doi: 10.3389/fpls.2021.708876 (PMC8415272; doi:10.3389/fpls.2021.708876)

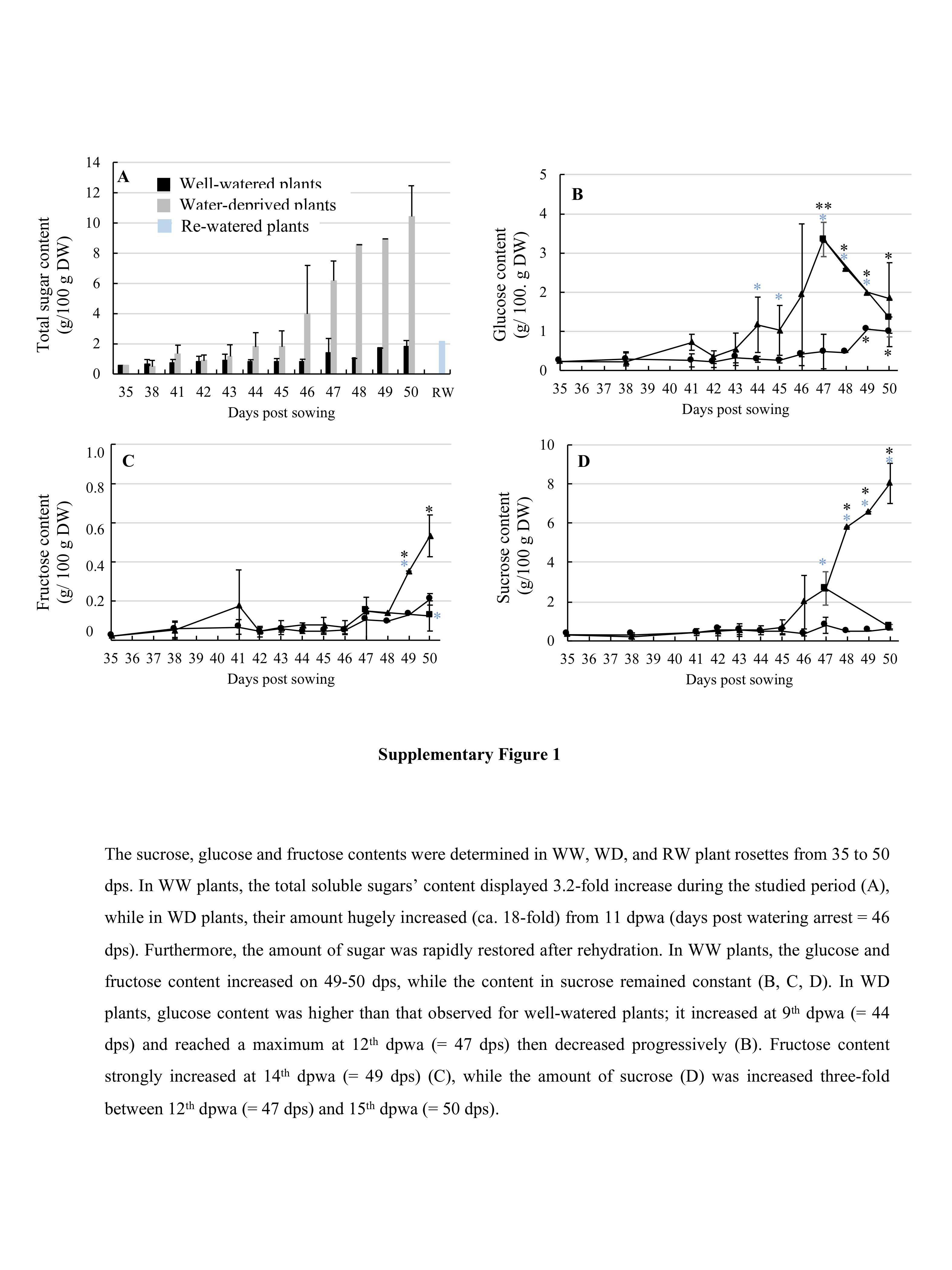

Supplement: Supplementary Figure 1 — Sugar content in the leaves of Arabidopsis thaliana Col-0. (A) Total sugar content, (B) glucose content, (C) fructose content, and (D) sucrose content at different days post-sowing. Well-watered (WW) (circle), water-deprived (WD; triangle), and re-watered (RW; square). The study was carried out with five plants per condition, and three independent biological repeats were performed (±SD). Dps, days post-sowing. The blue asterisks correspond to significant differences compared to the WW plants, determined by the Mann–Whitney test (p < 0.05). The black asterisks correspond to significant differences determined by the Kruskal–Wallis test, followed by Dunn's multiple comparisons test (*p < 0.05; ** p < 0.001). [file Image_1.JPEG]

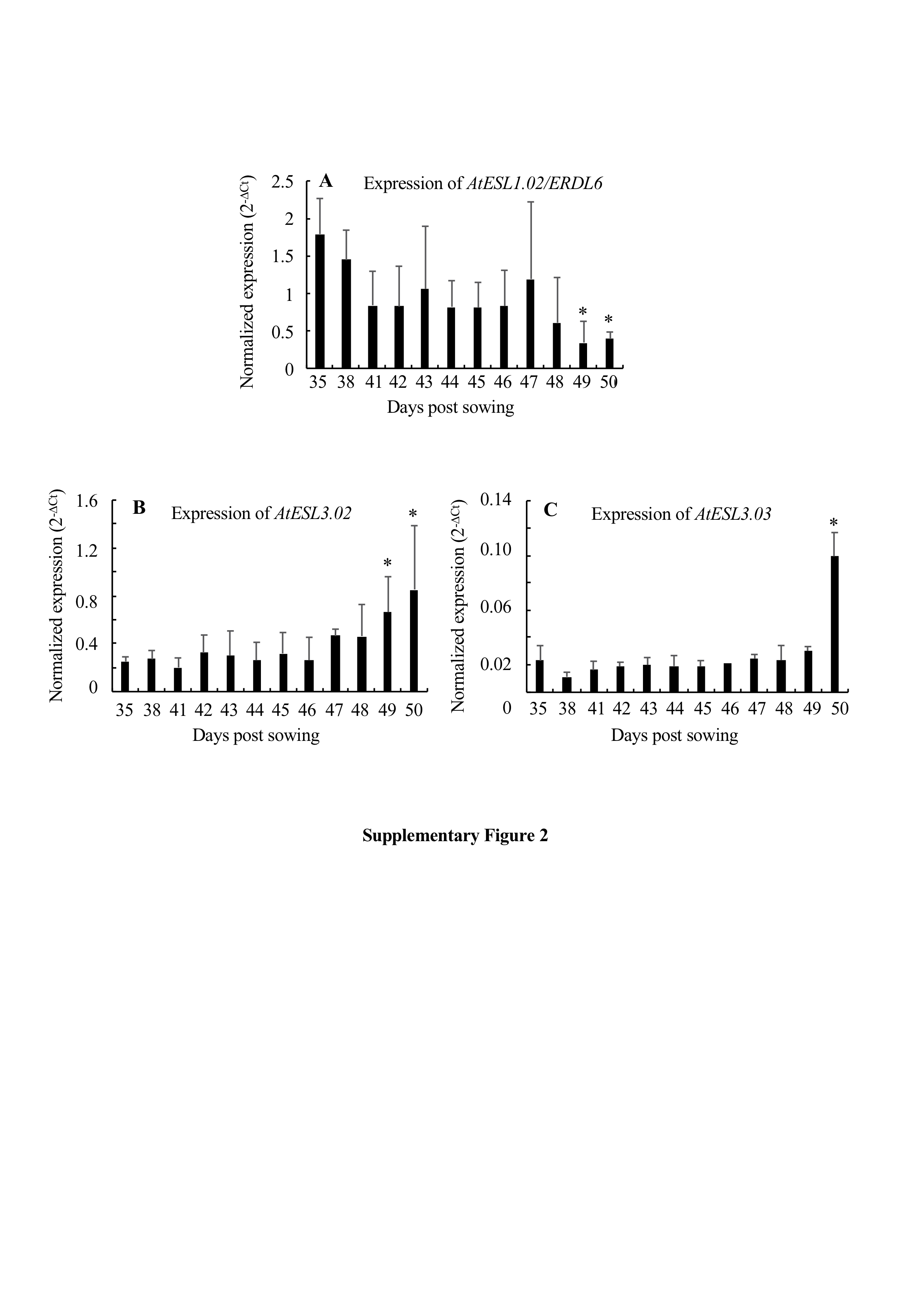

Supplement: Supplementary Figure 2 — Leaf relative expression of (A) AtESL1.02/ERDL6, (B) AtESL3.02, and (C) AtESL3.03 during the development of A. thaliana Col-0 under WW condition. 2−ΔCt values are normalized according to AtPP2a expression. The study was carried out on five plants and in three independent biological replicates. The asterisks represent the significantly different values determined by the Kruskal–Wallis test, followed by Dunn's multiple comparisons test (p < 0.05) with the Bonferroni correction. [file Image_2.JPEG]

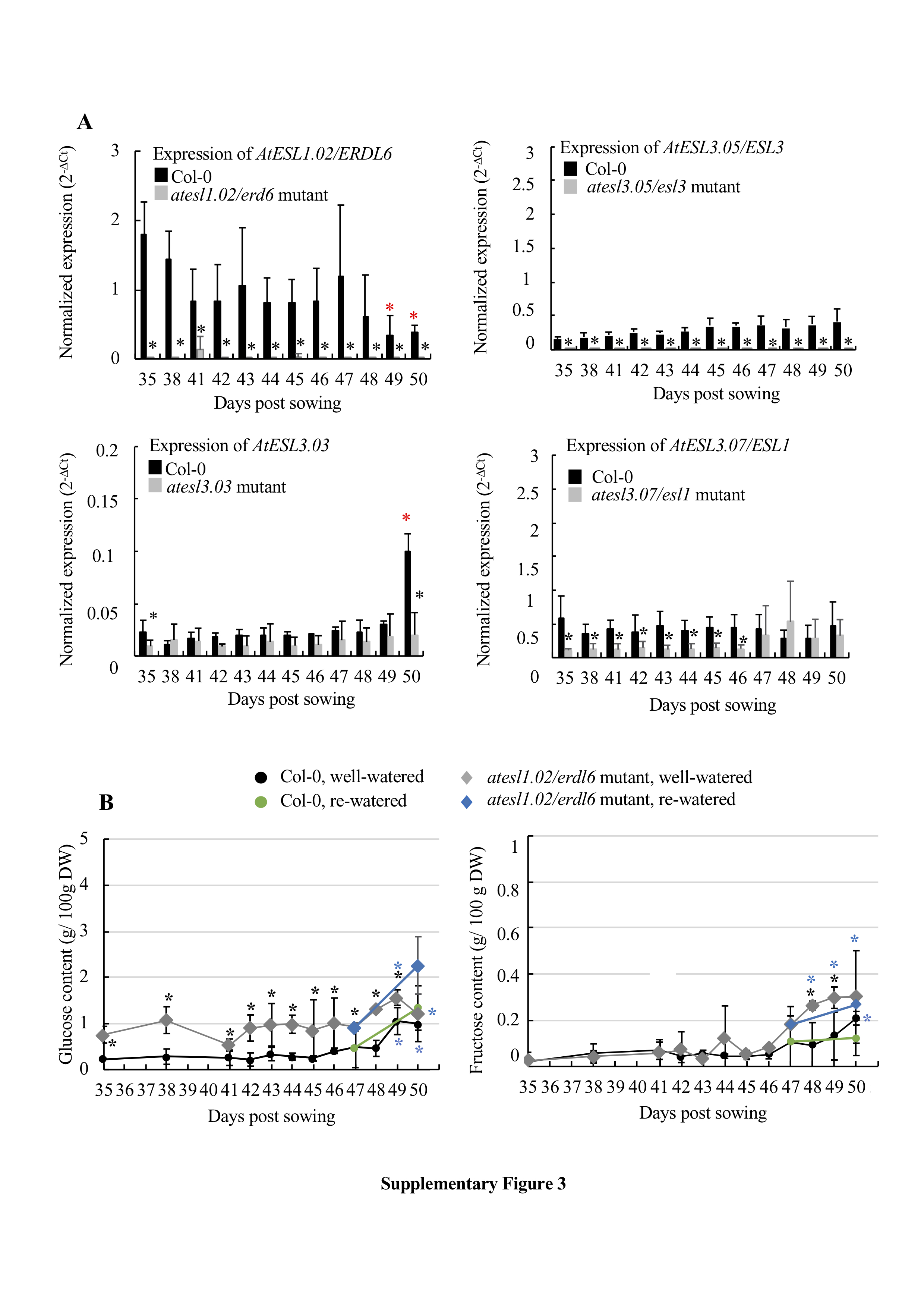

Supplement: Supplementary Figure 3 — (A) Expression level of AtESL1.02/ERDL6, AtESL3.03, AtESL3.05/ESL3, and AtESL3.07/ESL1 in the leaf of T-DNA insertional mutants: atesl1.02/erdl6, atesl3.03, atesl3.05/esl3, and atesl3.07/esl1. Comparison of the expression levels of the corresponding gene in its respective mutant and in the Col-0 during the growth kinetics in WW plants. The 2−ΔCt values are normalized according to AtPP2a expression. Black asterisks correspond to significant differences between atesl mutant and Col-0 plants, determined by the Mann–Whitney test (p < 0.05). Red asterisks correspond to significant differences determined by the Kruskal–Wallis test, followed by a Dunn's test for multiple comparisons with the Bonferroni correction (p < 0.05). (B) Glucose and fructose contents in the leaf of A. thaliana Col-0 and atesl1.02/erdl6 mutant during the growth kinetics in WW plants and after RW plants. Each value represents the mean of three biological repeats (±SD). Blue asterisk correspond to statistically different values along the kinetics, determined by the Kruskal–Wallis test (p < 0.05) followed by the Dunn's multi-comparison test. Black asterisks correspond to significant differences between atesl1.02/erdl6 mutant and Col-0 plants, determined by the Mann–Whitney test (p < 0.05). [file Image_3.JPEG]

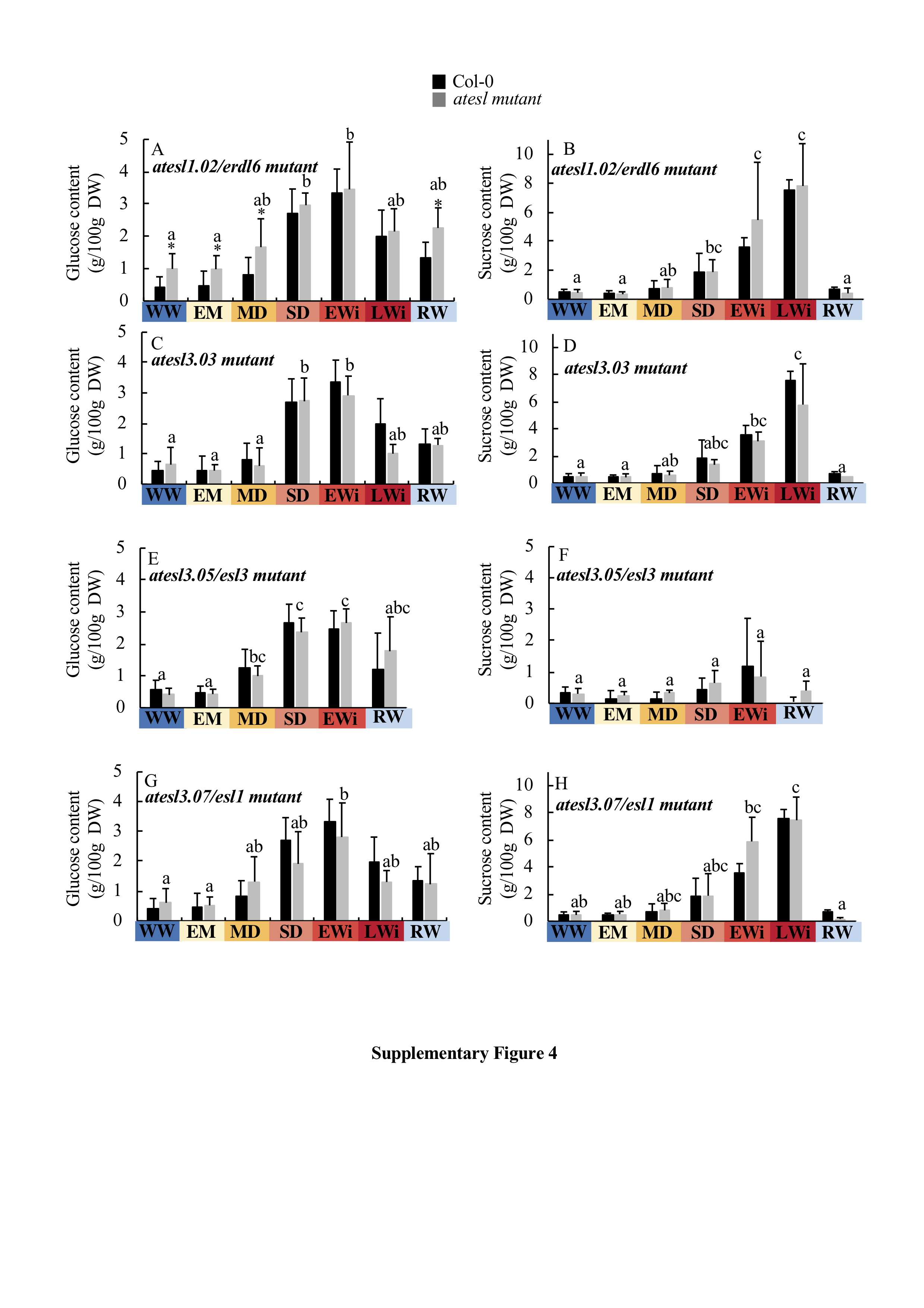

Supplement: Supplementary Figure 4 — Glucose and sucrose content in leaves of A. thaliana of Col-0 and the atesl mutants (1.02/erdl6, atesl3.03, atesl3.05/esl3, and atesl3.07/esl1) grown under WW, WD, and RW plants. Sugar contents are presented as a function of the four water-deficit phases defined according to leaf water content (WC%). WW, well-watered; EM, early mild water deficit; MD, moderate water deficit; SD, severe water deficit; EWi, early wilting; LWi, late wilting. Statistical analysis was performed using the Kruskal–Wallis (p < 0.05) test and Mann–Whitney pairwise comparison test (p < 0.05). Significantly different values are indicated by distinct letters and by an asterisk, respectively. [file Image_4.JPEG]
