# Supplementary material for: Responsiveness of Early Response to Dehydration Six-Like Transporter Genes to Water Deficit in Arabidopsis thaliana Leaves
Source: Front Plant Sci. 2021 Aug 16;12:708876. doi: 10.3389/fpls.2021.708876 (PMC8415272; doi:10.3389/fpls.2021.708876)
Supplement: Supplementary Figure 5 — Characterization of the effect of water deficiency on the Arabidopsis A. thaliana Col-0 and the atesl mutants (1.02/erdl6, atesl3.03, atesl3.05/esl3, and atesl3.07/esl1) grown under WW, WD, and RW plants. (A: a) PLA: projected leaf area, (B: b) FW: fresh weight, (C:c) DW: dry weight, (D: d) TW: turgid weight, (E: e) RWC: relative water content, (F: f) WC: water content, (G: g) SC: stomatal conductance. Col-0 (circle), atesl1.02/erdl6 (triangle), atesl3.03 (diamond), atesl3.07/esl1 (square), and atesl3.05/esl1 (cross). The study was carried out with five plants per condition, and three independent biological repeats were performed (±SD). Statistical analysis was performed using the Mann–Whitney pairwise comparison test (p < 0.05). Significantly different values are indicated by asterisk. [file Presentation_1.PPTX]

## Slide 1
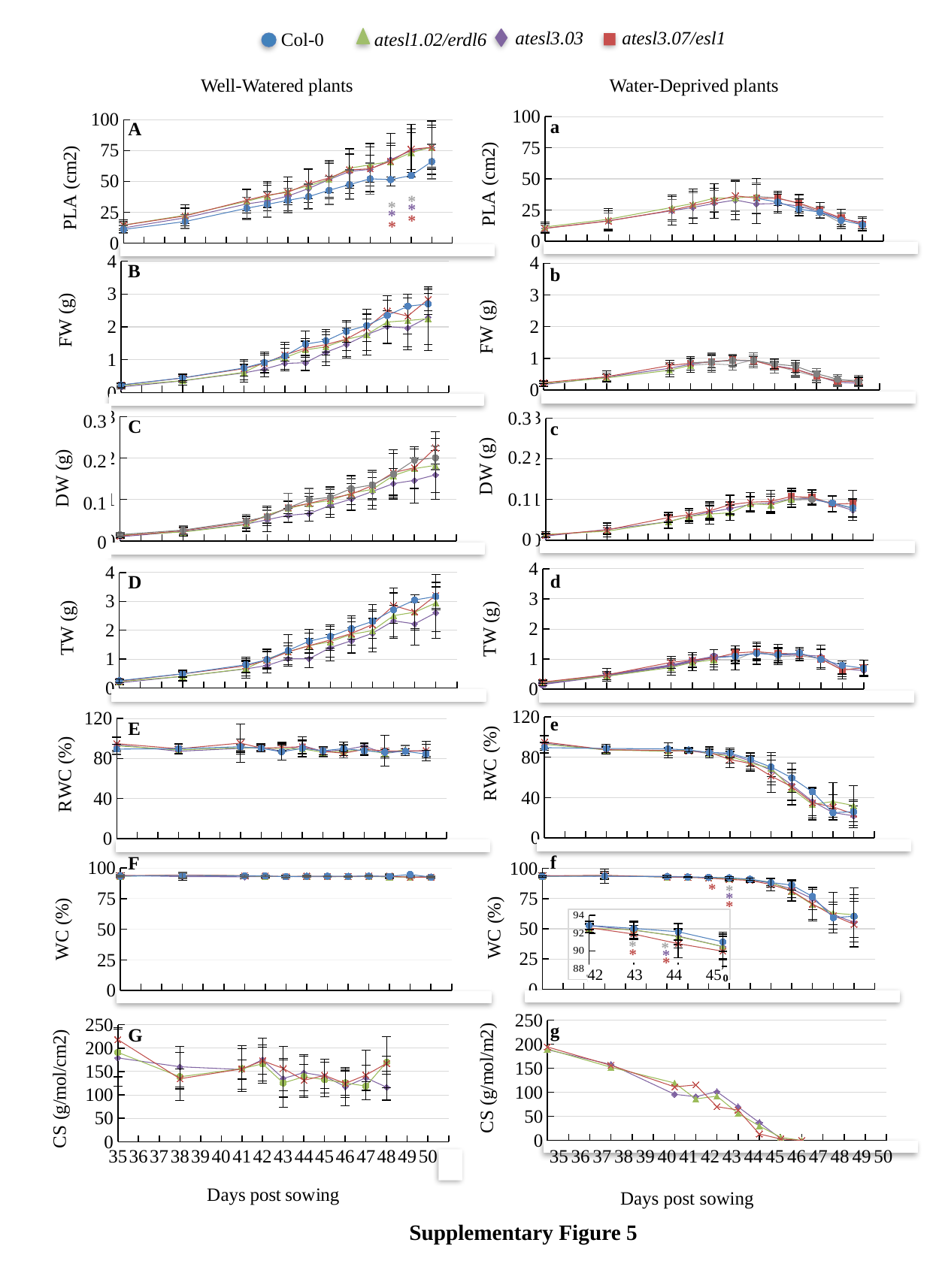

atesl3.03
atesl3.07/esl1
Col-0
atesl1.02/erdl6
Well-Watered plants
Water-Deprived plants
### Chart
| Category | | | | | | | | |
|---|---|---|---|---|---|---|---|---|
### Chart
| Category | | | | |
|---|---|---|---|---|a
A
*
*
*
*
*
*
### Chart
| Category | | | | |
|---|---|---|---|---|
### Chart
| Category | | | | | | | | | | | | |
|---|---|---|---|---|---|---|---|---|---|---|---|---|
B
b
### Chart
| Category | | | | | | | | | | | | |
|---|---|---|---|---|---|---|---|---|---|---|---|---|
### Chart
| Category | | | | |
|---|---|---|---|---|0.3
0.2
0.1
0
0.3
0.2
0.1
0
C
c
### Chart
| Category | | | | |
|---|---|---|---|---|
### Chart
| Category | | | | | | | | | | | | |
|---|---|---|---|---|---|---|---|---|---|---|---|---|d
D
### Chart
| Category | | | | |
|---|---|---|---|---|
### Chart
| Category | | | | |
|---|---|---|---|---|e
E
f
F
### Chart
| Category | | | | |
|---|---|---|---|---|
### Chart
| Category | | | | |
|---|---|---|---|---|*
*
*
*
### Chart
| Category | | | | |
|---|---|---|---|---|*
*
*
*
*
42 43 44 45
35 36 37 38 39 40 41 42 43 44 45 46 47 48 49 50 51
### Chart
| Category | | | | |
|---|---|---|---|---|
### Chart
| Category | | | | |
|---|---|---|---|---|g
G
35 36 37 38 39 40 41 42 43 44 45 46 47 48 49 50
Days post sowing
Supplementary Figure 5

## Slide 2
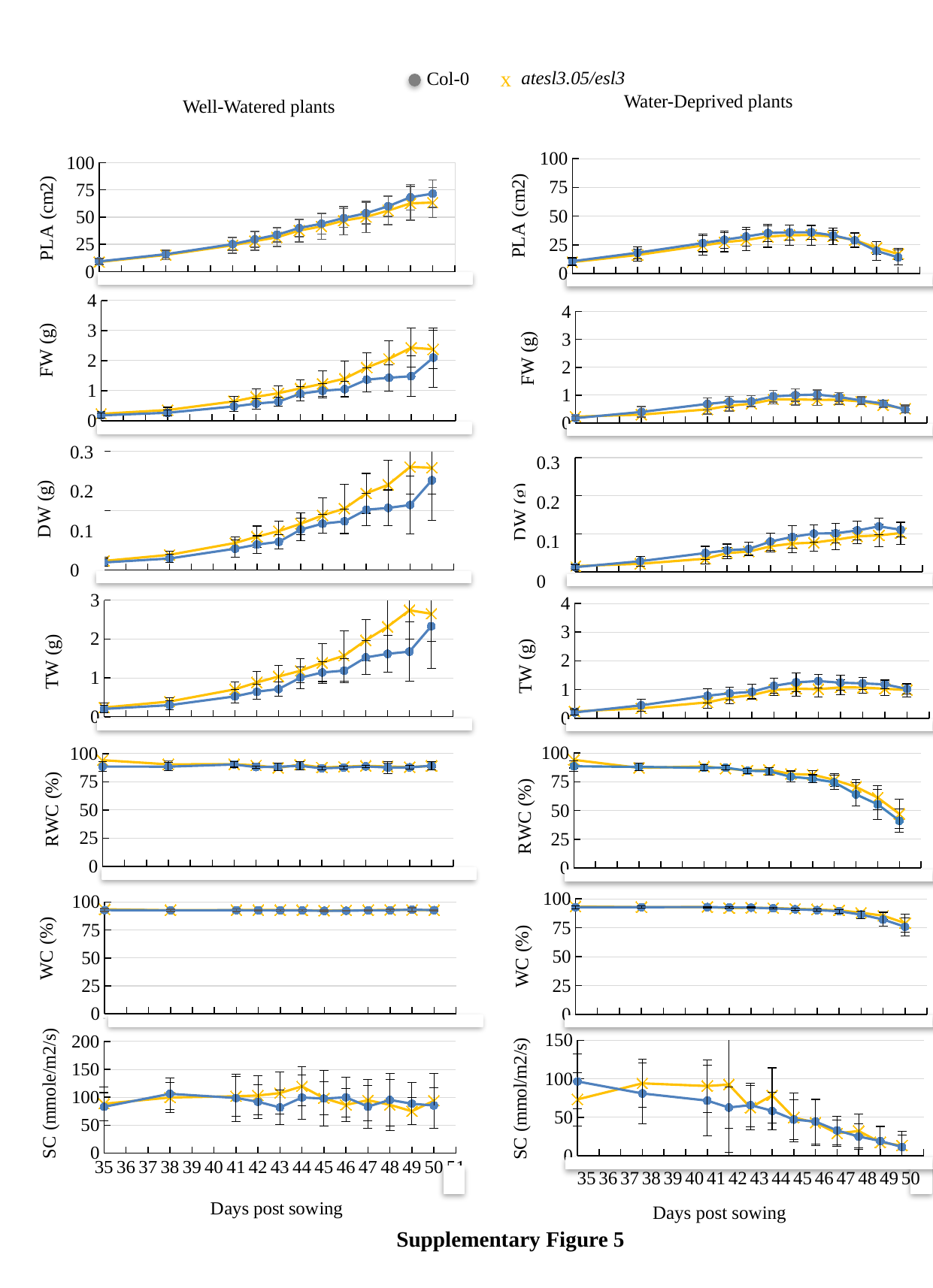

x
atesl3.05/esl3
Col-0
### Chart
| Category | Col-0 | esl3 |
|---|---|---|
### Chart
| Category | Col-0 | esl3 |
|---|---|---|
### Chart
| Category | Col-0 | esl3 |
|---|---|---|
### Chart
| Category | Col-0 | esl3 |
|---|---|---|0.3
0.2
0.1
0
0.3
0.2
0.1
0
### Chart
| Category | Col-0 | esl3 |
|---|---|---|
### Chart
| Category | Col-0 | esl3 |
|---|---|---|
### Chart
| Category | Col-0 | esl3 |
|---|---|---|
### Chart
| Category | Col-0 | esl3 |
|---|---|---|
### Chart
| Category | Col-0 | esl3 |
|---|---|---|
### Chart
| Category | Col-0 | esl3 |
|---|---|---|WC (%)
### Chart
| Category | Col-0 | esl3 |
|---|---|---|
### Chart
| Category | Col-0 | esl3 |
|---|---|---|
35 36 37 38 39 40 41 42 43 44 45 46 47 48 49 50
Days post sowing
Water-Deprived plants
Well-Watered plants
### Chart
| Category | Col-0 | esl3 |
|---|---|---|
### Chart
| Category | Col-0 | esl3 |
|---|---|---|
Supplementary Figure 5
