# Supplementary material for: Responsiveness of Early Response to Dehydration Six-Like Transporter Genes to Water Deficit in Arabidopsis thaliana Leaves
Source: Front Plant Sci. 2021 Aug 16;12:708876. doi: 10.3389/fpls.2021.708876 (PMC8415272; doi:10.3389/fpls.2021.708876)
Supplement: Supplementary Table 1 — Primers used in quantitative PCR (qPCR) for gene expression analysis and used for genotyping. [file Table_1.DOCX]

Supplementary Table 1: Primers used in qPCR for gene expression analysis and primers used for genotyping.

| **Gene** | **Accession** | **Gene expression**  **Sequence 5'-3'** | **Salk** | **Genotyping**  **Sequence 5'-3'** |
| --- | --- | --- | --- | --- |
| *ESL1.01* | At1g19450 | F : TAAGCCTCGTGATTGTAGCA | **-** | **-** |
|  |  | R : CAAGAAATAACCATAGCCACA |  |  |
| *ESL1.02/ERDL6* | At1g75220 | F : GTGGAGGAACTTTCACTCTGTA | SALK_106049 | LP: GCAACAAATCCAAGATTCGAC |
|  |  | R : GTTCTTCAAGAGTTTTGCCTT |  | RP: TATCCCCAAGTTTTGTTTCC |
| *ESL2.01/ZIF2* | At2g48020 | F : CTGAGATATTTCCCATAAACAT | **-** | **-** |
|  |  | R : AAGATTGCAATTTATGTCCCAG |  |  |
| *ESL2.02* | At3g05150 | F : TTCTTTTGAAGGCTCATGGTT | **-** | **-** |
|  |  | R : GACAAGCCATGAACTTAGCCA |  |  |
| *ESL2.03* | At5g18840 | F : CTGAGATATTTCCGATAAACGTA | **-** | **-** |
|  |  | R : TCTTCGCCACAAATATTATCGTC |  |  |
| *ESL3.01* | At1g54730 | F : AATCCGGCAGGAACGTTTTATG | **-** | **-** |
|  |  | R : CGTTATAATTAGGGACTGATAT |  |  |
| *EL3.02* | At4g04750 | F : TACAGGAACGTTTATGATGTTT | **-** | **-** |
|  |  | R : CATGAAATATAAAGAGATCTGA |  |  |
| *ESL3.03* | At4g04760 | F : CAGAGATTTATCCAGTAGATGT | SALK_132009 | LP : TTTACCGAAAGCACTCCATTG |
|  |  | R : TGGCTATGAACACAAATCCAA |  | RP : ATTGATCCCATACCAGAACC |
| *ESL3.04* | At3g20460 | F : ACGCTATGCAATTTAACTAGCTG | **-** | **-** |
|  |  | R : AACACACCAGAAGAGCTCCACT |  |  |
| *ESL3.05/ESL3* | At1g08890 | F : TGGAGTGCTTTCGGAACATATT | SALK_047351 | LP : TGCTCTTGCAACAATTTTGTG |
|  |  | R : GAGTAAAGAATCAACTTTGGAT |  | RP : TTTACCGAAAGCACTCCATTG |
| *ESL3.07/ESL1* | At1g08920 | F : TCGGTCTCATTCCATGTGCG | SALK_025646 | LP: AGAACCAAAACGACATCAACG |
|  |  | R : TGCATCATCTCCGCGAAGAC |  | RP : TCCCCATTTTCCCTATACACC |
| *ESL3.08/ERD6* | At1g08930 | F : ATAATGGCTGAGATATTTCCG | **-** | **-** |
|  |  | R : ATAAATACGATCGAACTGGC |  |  |
| *ESL3.10* | At3g05400 | F : CTTCTTATGACTTCAGCGTTGG | **-** | **-** |
|  |  | R : CAATTGCGAACATCATCGT |  |  |

| *ESL3.11* | At3g05160 | F : GCACAAGGAACCTTTTACATAT | **-** | **-** |
| --- | --- | --- | --- | --- |
|  |  | R : AATTCTTCAAGCGACTGTCC |  |  |
| *ESL3.13/SFP1* | At5g27360 | F : TATGTCGGAGATATTTCCAATA | **-** | **-** |
|  |  | R : CAAATTAAGTTTGATTTCTTTC |  |  |
| *ESL3.14/SFP2* | At5g27350 | F : AGTGGAGCACTCAAGGAACATT | **-** | **-** |
|  |  | R : TTGATTTATTCTATCGGGCTC |  |  |
| *SWEET16* | At3g16690 | F : TGCTGAATCCATCTACGTTCTCA | **-** | **-** |
|  |  | R : ACAGAGTTCTTGTTCCCGCAA |  |  |
| *SWEET17* | At4g15920 | F: AGTGACAACAAAGAGCGTGAAATAC | **-** | **-** |
|  |  | R: ACTTAAACCGTTGCTTAAACCAACC |  |  |
| *SUC4* | At1g09960 | F: AGTGTCAAGCGAGGAACGCATA | **-** | **-** |
|  |  | R: AGTCACACGAGAAGCCATTGC |  |  |
| *VGT2* | At5g17010 | F : AACCATTTCACTTCAGTCGCC | **-** | **-** |
|  |  | R : ATGCACCGTAAAGTGAGCCA |  |  |
| *TMT1* | At1g20840 | F : GACAACGATTTGCGTAGCCC | **-** | **-** |
|  |  | R : CCATGCTACTTTCGCCGTTG |  |  |
| *TMT2* | At4g35300 | F : GTCGCCGTCCCATGCTAATA | **-** | **-** |
|  |  | R : GACCACAAGACCAACCCCAA |  |  |
| *βFRUCT3* | At1g62660 | F : GCAGTCTATTCCGGTTTGCT | **-** | **-** |
|  |  | R : CTCCGTCGCTATTTCAGCCTTG  (Yamada *et al*., 2010) |  |  |
| *βFRUCT4* | At1g12240 | F : TAGATCCAACGACGAAGGGATC | **-** | **-** |
|  |  | R : TCCGATCACCGGAAAGTT  (Yamada *et al*., 2010) |  |  |
| *PP2a* | At1g13320 | F: TAACGTGGCCAAAATGATGC | **-** | **-** |
|  |  | R: GTTCTCCACAACCGCTTGGT  (Czechowski *et al.*, 2005) |  |  |
|  |  |  | LBb1.3 | ATTTTGCCGATTTCGGAAC |
